# Stability of cellulase in ionic liquids: correlations between enzyme activity and COSMO-RS descriptors

**DOI:** 10.1038/s41598-019-53523-5

**Published:** 2019-11-25

**Authors:** Jacob Nedergaard Pedersen, Bianca Pérez, Zheng Guo

**Affiliations:** 1 Department of Engineering, Faculty of Science and Technology, Aarhus University, 8000 Aarhus, Denmark; 2 Center for Food Technology, Danish Technological Institute, 8000 Aarhus, Denmark

**Keywords:** Enzymes, Hydrolases

## Abstract

Ionic liquids (ILs) are effective in pretreating cellulose for enhanced enzymatic saccharification, however ILs can inactivate cellulases. To guide the selection of ILs, the activity of cellulase was correlated with COSMO-RS calculations and descriptors of ILs including hydrogen bond (H-bond) basicity/acidity, polarity and ion size. Trends were deduced using an anion-series and a cation-series of ionic liquids in aqueous solutions. The activity in the cation-series was best correlated with the size of varied cations, whereas the activity in the anion-series showed a pronounced correlation to H-bond basicity and polarity of different anions. COSMO-RS was further used to predict the solubility of cellulose in ILs, which was correlated with cellulase activity on IL-pretreated cellulose. The best correlations were found between the enzyme activity in the anion-series ILs and the logarithmic activity coefficients, the H-bond energy, H-bond basicity and polarizability, underlining that the anion plays a crucial role in cellulose dissolution.

## Introduction

Lignocellulosic biomass, originating from wood or agricultural residues such as corncobs and wheat straw, is a promising renewable carbon source for the production of second-generation bioethanol or fine-chemicals^1,2^. However, the inherent recalcitrant nature of lignocellulosic biomass, due to the complex interactions between cellulose, lignin and hemicellulose, limits the enzymatic hydrolysis of cellulose into mono- and disaccharides^3^. Currently, a pretreatment of the biomass is needed to increase the saccharification yields during enzymatic hydrolysis. One such pretreatment method is the use of ionic liquids (ILs), which have attracted a great deal of interest, due to their ability to solubilize cellulose and their perception as green designer solvents^3–7^.

ILs’ ability to dissolve cellulose is mainly attributed to the anions’ H-bond acceptor capabilities i.e. high H-bond basicity with the ability to form strong H-bonds with the equatorial hydroxyl groups in cellulose^8^. Small anions with high H-bond basicities such as halogens, carboxylates and dialkyl phosphates are particular good at dissolving cellulose^3,9^. The best cations for dissolving cellulose have been reported mainly to be imidazolium and pyridinium based ILs, which is attributed to their aromatic nature and their lower interaction strengths with the paired anion^10^. The attractive properties of ILs cellulose dissolution are cancelled out by the inactivation of cellulases caused by the ILs^11,12^. One approach to avoid the IL-mediated inactivation of cellulases, is to precipitate the treated biomass from the IL using water. However, residual IL concentrations can still exceed 10–15% (v/v), which is sufficient to inactivate the cellulases^11^. In a comparison study between some of the most common cellulose-dissolving ILs ([DMIM][DMP], [BMIM][Cl], [EMIM][Oac] and [AMIM][Cl]), cellulase activity decreased up to 70–85% compared to buffer in the presence of 10% (v/v)IL^13^.

The IL-induced inactivation of cellulases is caused by a variety of physiochemical properties of the ILs^12^. It has been reported that the inactivation is mainly caused by anions with high H-bond basicity, as nucleophilic anions can coordinate to positively charged surface residues and cause conformational changes of the cellulases leading to denaturation^11,14,15^. Molecular dynamic simulations have shown that [BMIM]-cations initialized through their hydrophobic tails, are able to intrude into the binding tunnel of cellobiohydrolase I from *Trichoderma reesei* and bind at the active site inactivating the enzyme^16^. Molecular dynamic simulations have also revealed that the effects of ILs vary among different cellulases from local structural disturbances to loss of secondary structure^17^.

As the combinations of possible ILs with different cations and anions are numerous, a rapid and effective screening method is needed to find those ILs that have good cellulose dissolving capabilities but only to a lesser extent inactivate the cellulases. The computational model COSMO-RS (Conductor-like Screening Model for Real Solvents) integrates dominant interactions such as electrostatic (polarity), H-bonds, van der Waals interactions and misfits to summarize multiple solvation interactions of ILs and can be used to predict several properties of liquid mixtures^18^. Previous publications have demonstrated that COSMO-RS can predict ILs’ cellulose dissolving capabilities. Liu *et al*.^19^ used COSMO-RS to screen the combination of 17 cations and 21 anions for their ability to dissolve cellulose using the logarithmic activity coefficients as an indirect measure of cellulose solubility. Out of three different models (glucose, mid-monomer part of cellotriose and mid-dimer part of cellotetraose), the mid-monomer part of cellotriose was found to be closer to the experimental results. The dissolution process was largely anion-dependent and excess enthalpy calculations indicated that the main driving force in the cellulose dissolution could be attributed to H-bonding between cellulose and the IL. Kahlen *et al*.^20^ calculated the logarithmic activity coefficients of the mid-monomer part of cellotriose in more than 2200 ILs using COSMO-RS and found that the anion played a crucial role in contributing to the dissolving power of an IL. Finally, attempts have been made to correlate enzyme activity, mostly for lipases, with COSMO-RS calculations and descriptors such as solubility calculations, logarithmic activity coefficients, σ-profiles and interaction energies^21–24^. Thus, COSMO-RS can both predict ILs ability to dissolve cellulose and can provide useful physiochemical information about ILs that might be able to explain observed enzyme activities. Unfortunately, no reports exist with respect to the correlation of cellulase activity to the solvent property/descriptors of IL-mediated systems.

For a successful saccharification using ILs as a pretreatment medium, it is crucial that the cellulase is still active in the presence of reasonable amounts of IL. Using a one-pot, wash-free configuration that combines IL pretreatment and saccharification, 10–20% residual IL is considered to be economically sustainable when accounting for costs associated with water usage and energy-intensive evaporation^25^. Hence, an IL should be able to dissolve high amounts of cellulose as as well as causing negligible enzyme inactivation at 10–20% IL levels. However, reports quantitatively correlating the structural features of ILs with apparent activity of cellulases are scarce. The objective of this work was to establish correlations between COSMO-RS descriptors (molecular size, H-bond acidity/basicity, polarity etc.) and cellulase activity using a commercial cellulase cocktail from *Trichoderma reesei* in the presence of different ILs with and without pretreatment of the cellulose.

## Results and Discussion

### COSMO-RS screening of ionic liquids

**Figure 1:**
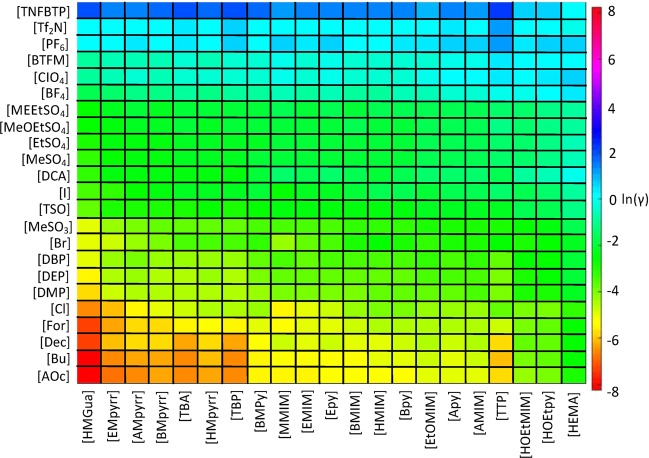
COSMO-RS predicted logarithmic activity coefficients of the mid-monomer of cellotriose in 483 ILs at infinite dilution. The calculation temperature was 90 °C. Tabulated values of the predicted logarithmic activity coefficients can be found in the supporting information (Fig. S2).

COSMO-RS was used to predict the logarithmic activity coefficients, ln(γ) (Fig. 1), of the midmonomer part of cellotriose (Supplementary Fig. S1) at infinite dilution using a combination of 23 anions and 21 cations (Supplementary Tables S1 and S2) totaling 483 ILs. The mid-monomer part of cellotriose was selected in the calculations as this has previously been proved to be one of the best models to represent the cellulose polymer, whereas low ln(γ) have been proved to be related to higher cellulose solubilities^19,20^. The anions and cations are listed based on their predicted ln(γ), with ILs predicted to be good at dissolving cellulose are located in the bottom right corner of Fig. 1, while ILs predicted to be poor at dissolving cellulose are located in the top of Fig. 1. The logarithmic activity coefficient for different anions varied significantly, whereas the cation only had a minor effect. Anions based on carboxylates, [Cl] and dialkyl phosphates combined with cations such as [HMGua], pyrrolidium-based and [TBA] were predicted to be good ILs for cellulose dissolution due to their highly negative predicted logarithmic activity coefficients. The results are in good agreement with previous reported results^19,20^.

**Figure 2:**
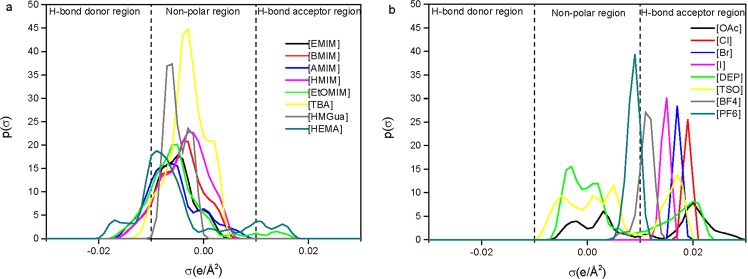
COSMO-RS generated σ-profiles of selected cations (**a**) and anions (**b**). All except [HEMA], [HMGua], [BF_4_] and [PF_6_] were included in the two series of ILs investigated.

Kahlen *et al*.^20^ explained that the interactions between cellulose and the IL have to go beyond the energy of the H-bonds (up to 25 kJ/mol) that holds the cellulose chains together. This can be obtained if the cation or anion is highly polar and the combination of the cation and anion is only slightly polar. This trend was confirmed by examining the σ-profiles of some selected cations and anions in Fig. 2. Both [OAc] and [Cl] are able to form H-bonds acting as acceptors as seen from their σ-profiles in Fig. 2, thus ILs containing these anions are predicted to be good at dissolving cellulose. In contrast, most of the σ-profile of [PF_6_] falls within the non-polar region, thus [PF_6_] cannot form hydrogen bonds with cellulose and dissolve it. [HMGua] was predicted to be the best cation, this might be due to the σ-profile of [HMGua] falls entirely within the non-polar region, confirming the criterion that having a slightly polar cation with a highly polar anion, should result in an IL with good cellulose dissolution capabilities. [HEMA] was predicted by COSMO-RS to be one of the worst cations. This ion is highly polar and will interact strongly with the anion thus hindering cellulose dissolution.

### Selection of ionic liquids and COSMO-RS descriptors

Based on the predictive screening of the ILs’ ability to dissolve cellulose and their corresponding COSMO-RS descriptors, different ILs were selected, including ILs predicted to be good or poor at dissolving cellulose, commercially available or well-studied ILs (Supplementary Table S3). The ILs were water-miscible and liquid at the 90 °C pretreatment temperature. The COSMO-RS descriptors are molecular descriptors that provide valuable information about the physics of the molecules^26^. Among the molecular descriptors are the second and third sigma moment, Sig2 and Sig3, which represent the polarity and polarizability of the molecule, respectively, with higher values indicating a more polar molecule or higher polarizability. The H-bond moments, Hb_acc3 and Hb_don3, correspond to the H-bond basicity and H-bond acidity of the molecule, respectively. Moreover, the size (area and volume) of the IL ions can be extracted from the COSMO-RS files^18,26^. The logarithmic activity coefficient of water in the different ILs were also predicted using COSMO-RS and used as an indirect measurement of water activity^27^. As the cation and the anion are treated as two separate ionic species in the COSMO-RS calculations, ILs containing either the same cation or anion will have the same ion specific descriptors such as same Hb_acc3 for the anion and same Hb_don3 for the cation. Anions such as [Cl] and [OAc] do not contain any H-bond donor moieties hence they have an Hb_don3 of zero. Similar, cations such as 1-alkyl-3-methylimidazolium-based cations do not contain any H-bond donor moieties, thereby having an Hb_acc3 of zero. Therefore, two different series were investigated among the tested ILs (Table 1), namely an anion-series consisting of ILs with different anions but all containing the [EMIM]-cation and a cation-series consisting of different cations but all containing the [Cl]-anion. The series were used to decipher trends of correlation between the enzyme activity and the different COSMO-RS descriptors.

**Table 1:** The two IL-series studied in this work.

Anion-series		Cation-series	
#	IL	#	IL
1	[EMIM][Br]	1	[AMIM][Cl]
2	[EMIM][Cl]	2	[BMIM][Cl]
3	[EMIM][DEP]	3	[EMIM][Cl]
4	[EMIM][I]	4	[HMIM][Cl]
5	[EMIM][OAc]	5	[Apy][Cl]
6	[EMIM][TSO]	6	[EtOMIM] [Cl]
		7	[TBA][Cl]

### Correlation between enzyme activity and COSMO-RS descriptors in the presence of ILs

**Figure 3:**
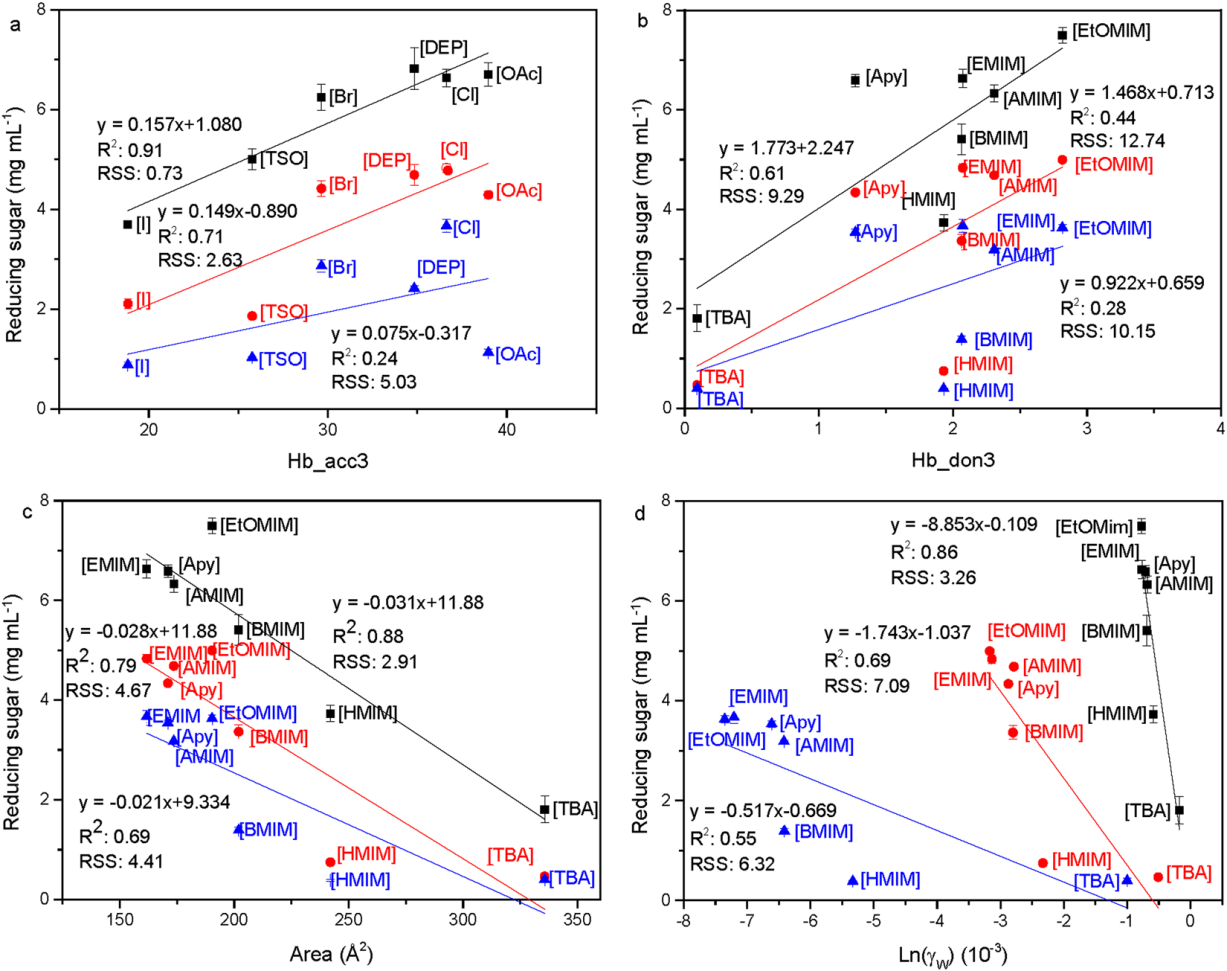
Reducing sugar released (mg mL^−1^) after 24 h of saccharification at 250 mM (■), 500 mM (●) and 750 mM (▲) IL versus COSMO-RS molecular descriptors: (**a**) Hb_acc3 for the anion-series, (**b**) Hb_don3 for the cation-series, (**c**) Area (Å^2^) of the cation-series, (**d**) logarithmic water activity of the cation-series. Experimental conditions: Avicel cellulose loading = 2.5% (w/v), cellulase loading = 1 mg mL^−1^, pH = 5, T = 50 °C.

To investigate the correlation between cellulase activity on untreated cellulose and the COSMO-RS descriptors of the two IL series, the saccharification reactions were carried out at 250 mM, 500 mM and 750 mM IL (Supplementary Fig. S3). The amount of reducing sugars released were plotted against different COSMO-RS descriptors of the two IL-series and are shown in Fig. 3 and Supplementary Fig. S4.

Overall, the correlations (smaller R^2^-values) became worse with increasing concentrations. The plot of H-bond basicity (Hb_acc3) versus the amount of released sugars (Fig. 3a) indicated that anions with high H-bond basicities resulted in higher enzyme activities compared to anions with lower H-bond basicities. However, the amounts of released sugars were higher in the presence of [Cl], [Br] and [DEP] than in the presence of [OAc] at 500 mM and 750 mM. This might be attributed to the high H-bond basicity of [OAc] causing a destabilizing of the enzyme resulting in a lower activity compared to e.g. [EMIM][DEP] that has been reported to be more cellulase-friendly than [EMIM][OAc]^28^. The correlation between H-bond acidities versus the enzyme activity for the cation-series (Fig. 3b) were generally worse than that of the H-bond basicities for the anion-series. Sig2 (polarity) correlated positively with the enzyme activities for both the anion- and cation-series (Supplementary Fig. S4).

The cation-series showed a good linear fit between activity and the descriptors related to area (Fig. 3c) and volume (Supplementary Fig. S4) with larger cations causing lower enzyme activities. The cations interact with enzymes mostly through van der Waals interactions, hence large cations will interact stronger with the enzyme than smaller ones, which can lead to protein conformational change and deactivation. In addition, it has been shown that long alkyl-chains have a larger destabilizing effect compared to short alkyl-chains^12,29^. This was reflected for imidazolium cations for which the activity decreased in the following order: [EMIM] ≈ [AMIM] > [BMIM] > [HMIM]. However, there were found no correlation between descriptors related to size and the different anions in the anion-series (Supplementary Fig. S4). Suggesting that the hydrogen bond basicity of the anions have a greater impact on the enzyme activity than their sizes. This be related to the fact that the size of anions is generally much smaller compared to cations.

The logarithmic activity coefficient can be used as an indirect measurement of the water activity, which indicates the active water content around enzymes and is important for enzyme activity^30^. A negative correlation was observed between the predicted logarithmic activity coefficient of water and the enzyme activities of the cation-series (Fig. 3d), indicating that IL mixtures with lower ln(γ_w_) and hence a lower water activity, resulted in higher enzyme activities. This might be ascribed to a higher restrain of water caused by the higher solvation (lower ln(γ_w_)) of the cations with a smaller size but higher average positive density, such as [EMIM] > [BMIM] > [HMIM] (Fig. 3d).

The surface charge of cellulases plays an important role in maintaining the activity in the presence of ILs, where a high number of charged residues tends to stabilize cellulases in ILs^12,15^. As the interaction between the anion of ILs and the enzyme plays a major role in the denaturation of cellulases, it has been shown that a high number of acidic residues on the surface increase the stability of a cellulase cocktail from *T. reesei* in aqueous solutions of ILs through a tighter binding of the cation and preferential exclusion of the anion^11^. The commercial cellulase cocktail used in this study is composed primarily of the two exoglucanases Cel7A (up to 60%) and Cel6A (15–20%), which contain approximately 11 primary amines and 33 acid groups each and the two endoglucanases Cel7B (up to 10%) and Cel5A (up to 10%), which contain approximately 11 primary amines and 33 acid groups each. Together, these enzymes make up approximately 95% of the total enzyme content in the cocktail (Seiboth, 2011, Nordwald 2014). The majority of the charged residues are located on the surface. Given the mixed surface charge of the enzymes in the cellulase mixture, it is possible for the anions to interact with the positive charges of the enzyme causing disruption of e.g. salt-bridges leading to inactivation of the enzymes. Whereas the cations with long alkyl chains can interaction with non-polar surface patches or negatively charged residues near the active site, inactivating the enzyme. In addition, the carbohydrate binding module is believed to be particular sensitive towards ILs, which can also explain the decreased enzyme activity at higher concentrations^31^.

### Cellulase activity on IL-pretreated cellulose

**Figure 4:**
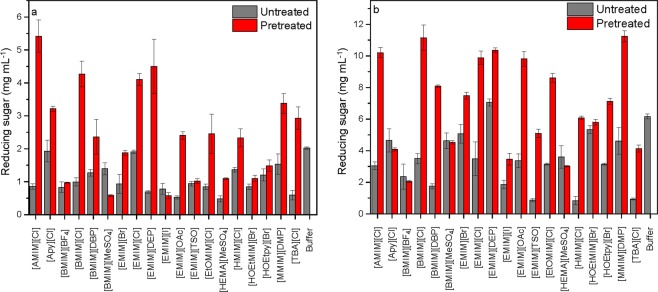
Reducing sugar (mg mL^−1^) measured after 1 h (**a**) and 24 h (**b**) of saccharification on untreated and pretreated cellulose respectively.10% w/w Avicel cellulose was pretreated in pure ILs prior to saccharification, then diluted in buffer to 9% w/v IL and 1% w/v cellulose. Untreated Avicel cellulose was saccharified in the presence of 9% w/v IL and 1% w/v cellulose. The cellulase loading was 1 mg mL^−1^, T = 50 °C.

To investigate the relationships between COSMO-RS calculations and descriptors versus the enzyme activity, the saccharifications were carried out on cellulose, which had been pretreated in 19 ILs at 90 °C for 24 h. The ILs included the ILs from the two series plus additional ILs. Prior to hydrolysis, the IL-cellulose mixtures were diluted in buffer, hence the enzymatic saccharifications were performed in diluted ILs/buffer mixtures. Thus, the effect on cellulase activity is a compromise between the enhanced accessibility of cellulase to cellulose and inactivation of the cellulase. The amounts of reducing sugars were measured after 1 h and 24 h as shown in Fig. 4. After 1 h of saccharification (Fig. 4a), the catalytic efficiency of cellulase was four to five times higher on cellulose which had been pretreated in ILs such as [AMIM][Cl], [BMIM][Cl] and [EMIM][DEP] compared to their untreated counterparts. Whereas, several ILs such as [BMIM][BF_4_], [EMIM][I] and [EMIM][TSO] did not show any significant differences between pretreated and untreated samples.

After 24 h (Fig. 4b) [AMIM][Cl], [BMIM][Cl], [EMIM][Br], [EMIM][DEP], [EMIM][OAc] and [MMIM][DMP] had reached complete saccharifications (10 mg mL^−1^). While ILs such as [BMIM][BF_4_], [BMIM][MeSO_4_] and [HEMA][MeSO_4_] showed almost no differences in the amount of reducing sugar released between the pretreated and non-pretreated cellulose in addition to low yields. This corresponds with their low ability to solubilize cellulose as predicted by the logarithmic activity coefficients (Fig. 1).

For the untreated samples, large differences were observed in the enzyme activities. The amount of reducing sugar after 24 h was 7 mg mL^−1^ in the presence of [EMIM][DEP], but around 3 to 4 mg mL^−1^ reducing sugar in the presence of [AMIM][Cl], [BMIM][Cl], [EMIM][Cl] and [EMIM][OAc], although they all showed complete saccharification on pretreated cellulose (Fig. 4b). Similarly, after 24 h the reducing sugar level were comparable for cellulose pretreated in [HMIM][Cl] and [HOEtMIM] [Br]. On untreated cellulose the reducing sugar level was 6 times lower in the presence of [HMIM][Cl] than in the presence of [HOEtMIM][Br] which was comparable to the pretreated sample (Fig. 4b). This emphasizes that ILs like [EMIM][DEP] and [HoEtMIM][Br] are more cellulase-friendly.

### Influence of logarithmic activity coefficient

**Figure 5:**
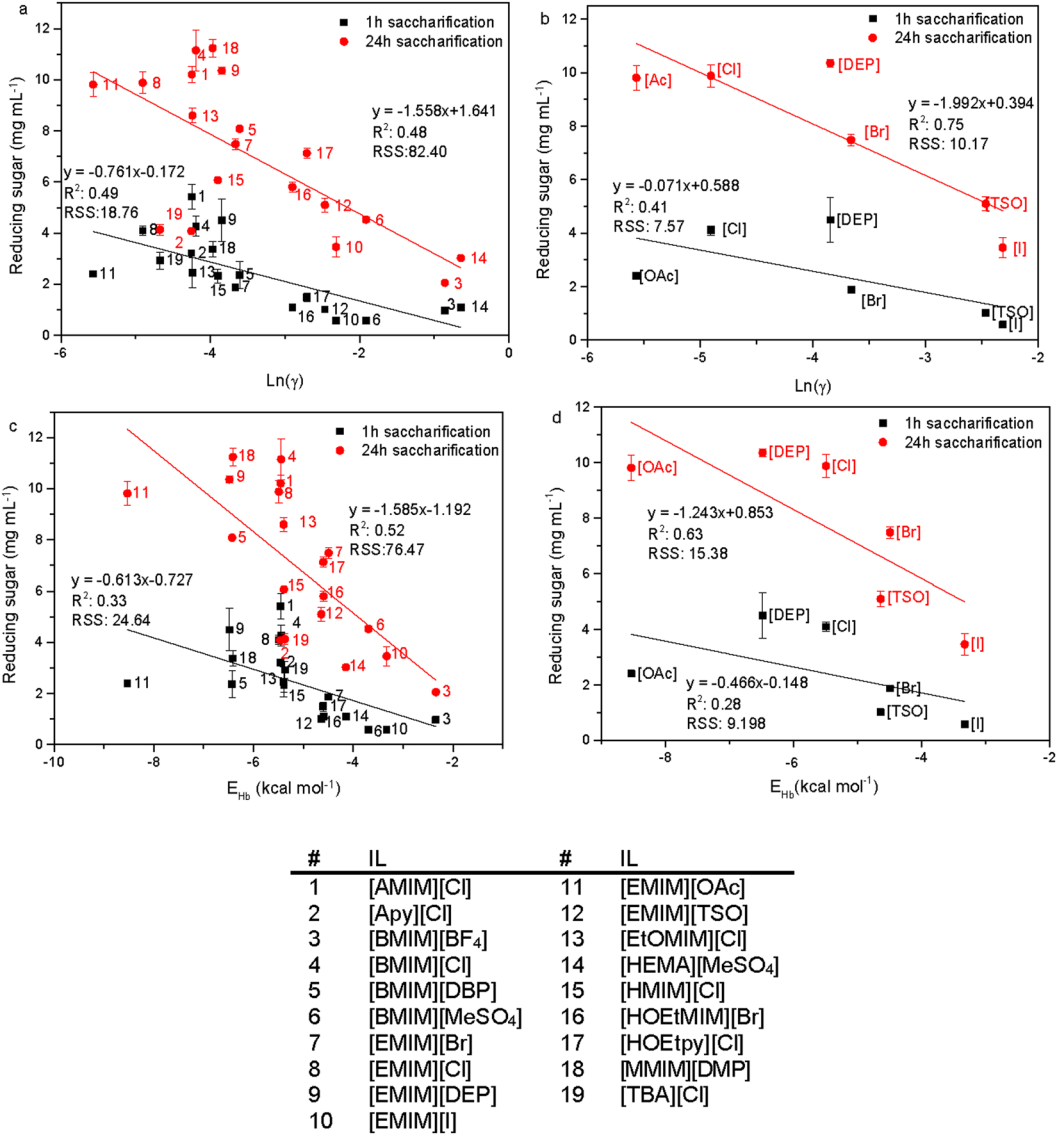
Reducing sugar (mg mL^−1^) measured after 1 h (■) and 24 h (●) of saccharification on IL-pretreated Avicel cellulose, versus: (**a**) the logarithmic activity coefficient for the19 ILs, (**b**) the logarithmic activity coefficient for the anion-series, (**c**) hydrogen bonding interaction energy (kcal mol^−1^) between the 19 ILs and the cellulose model, (**d**) hydrogen bonding interaction energy (kcal mol^−1^) between the anion-series and the cellulose model. The numbers corresponding to the ILs are specified in the table. For all the calculations, the mid-monomer part of cellotriose was used as the cellulose model.

The plot of the reducing sugars versus the logarithmic activity coefficients of the cellulose model in the 19 ILs is shown in Fig. 5a. Cellulose pretreated in ILs with a more negative predicted ln(γ) generally resulted in higher enzyme activities and were observed both after 1 h (R^2^ = 0.49) and 24 h (R^2^ = 0.48) of saccharification. This corresponds with previous reports that ILs with a low ln(γ) are good at dissolving cellulose, and thus making the cellulose more susceptible towards enzymatic hydrolysis^19,20^. Complete saccharification with yields around 100% were observed for the cellulose pretreated in [EMIM][OAc], [EMIM][Cl], [AMIM][Cl], [BMIM][Cl], [EMIM][DEP] and [MMIM][DMP]. The ln(γ) varied from −5.57 for [EMIM][OAc] to −3.85 for[EMIM][DEP]. The cellulose loading was fixed at 10 wt% during the pretreatment, thus the full potential of the ILs was not explored, as some of the ILs have been shown to be able to dissolve larger quantities of cellulose^32^. As the reaction mixtures contained residual IL, the residual IL had an impact on the enzyme activities. Thus, the efficiency of the enzymatic saccharifications is a trade-off between an IL’s capability to dissolve cellulose as predicted by the ln(γ) and the degree of inactivation of the cellulase caused by the IL. This explains why the amount of reducing sugar in [TBA][Cl]-pretreated cellulose (Fig. 5a, #19) was lower compared to the ILs with similar ln(γ), as [TBA][Cl] strongly inactivates the cellulase (Supplementary Fig. S3). [Apy][Cl] (Fig. 5a, #2) also exhibits lower activity compared to what would have been expected from the ln(γ), the reason is less clear compared to [TBA][Cl], as [Apy][Cl] did not cause a significantly different enzyme inactivation compared to some of the other ILs (Supplementary Fig. S3). Removing these two ILs improved the fit to R^2^ = 0.76 at 24 h (Supplementary Fig. S8). The logarithmic activity coefficient versus the amount of reducing sugar for the anion and cation-series are depicted in Fig. 5b and Supplementary Fig. S7, respectively. The fit between ln(γ) and the enzyme activity was better for the anion-series with R^2^-values of 0.41 and 0.75 at 1 h and 24 h, respectively (Fig. 5b) than that of the cation-series with R^2^-values of 0.19 and 0.002 at 1 h and 24 h, respectively (Supplementary Fig. S7). This underlines that cellulose dissolution is highly anion-dependent.

**Figure 6:**
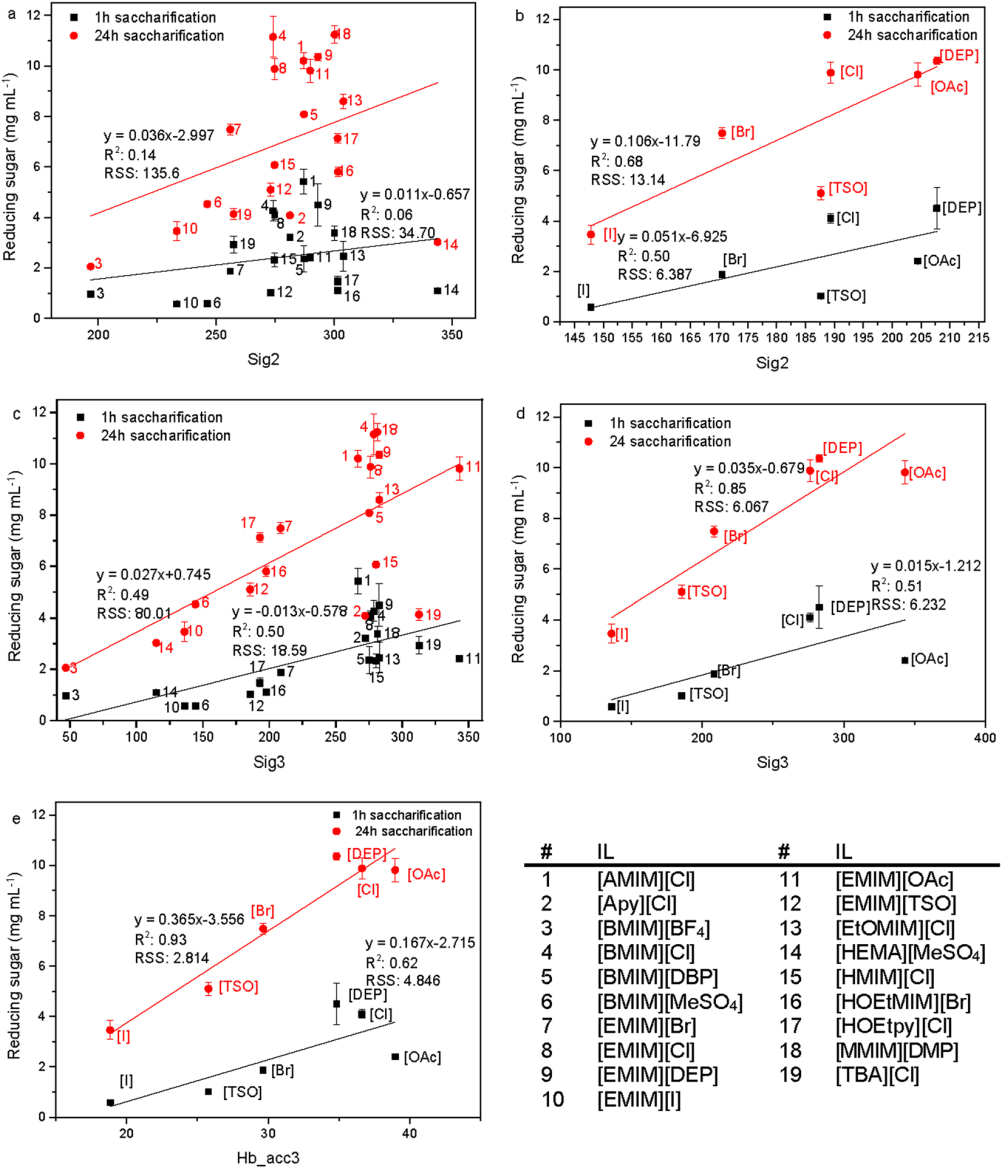
Reducing sugar (mg mL^−1^) measured after 1 h (■) and 24 h (●) of saccharification on IL-pretreated cellulose, versus: (**a**) Sig2 for all 19 ILs, (**b**) Sig2 for the anion-series, (**c**) Sig3 for all 19 ILs, (**d**) Sig3 for the anion-series, (**e**) H-bond basicity (Hb_acc3) for the anion-series. The numbers corresponding to the ILs are specified in the table.

Of the three interactions energies (H-bond, misfit and van der Waal), the most important was the contribution from H-bond interactions between the ILs and the cellulose model. It had the best linear fit with R^2^-values of 0.33 and 0.52 after 1 h and 24 h of saccharification, respectively (Fig. 5c). Omitting [Apy][Cl] (#2) and [TBA][Cl] (#19) at 24 h improved the fit to R^2^ = 0.63 at 24 h (Supplementary Fig. S8). This emphasizes that hydrogen bonding between cellulose and the IL is a key factor for the cellulose solubility making the cellulose more susceptible towards enzymatic hydrolysis, while the contribution from van der Waals forces (R^2^ = 0.02 at 24 h) and misfits (R^2^ = 0.17 at 24 h) are only secondary factors (Supplementary Fig. S5)^19^. For the cation- and anion-series, the latter showed the best fit between E_Hb_ and enzyme activity with R^2^ = 0.63 after 24 h (Fig. 6d) compared to a R^2^ = 0.23 for the cation-series (Supplementary Fig. S7). After only 1 h of saccharification, R^2^ of the cation-series was 0.45 compared to that of the anion-series of 0.28. The H-bond interaction energies in the cation-series are similar to each other, varying only from −5.37 kcal mol^−1^ for [TBA][Cl] to −5.49 kcal mol^−1^ for [EMIM][Cl], whereas the E_Hb_ in the anion-series varies from −3.80 kcal mol^−1^ for [EMIM][I] to −7.39 kcal mol^−1^ for [EMIM][OAc]. This highlight the fact that the anion plays a pivotal role in cellulose dissolution and hence enhanced enzyme activity compared to the cation.

### COSMO-RS descriptors and cellulase activity correlations

The correlations between enzyme activity and COSMO-RS descriptors are depicted in Fig. 6 and Supplementary Figs S5–S7. Cellulose pretreated in ILs with increasing polarity (Sig2) lead in general to higher enzyme activities (Fig. 6a,b) with the anion-series after 24 h showing the best correlation with the second sigma moment (R^2^ = 0.68). [HEMA][MeSO_4_] has the highest sig2 value (#14) and hence being the most polar IL, nevertheless the amount of reducing sugar was one of the lowest after 24 h (Fig. 6a). This can be attributed to the fact that this IL has a cation which is very polar thereby interacting more strongly with the anion resulting in the lowest ln(γ). Removing #14 improved the fit from R^2^ = 0.14 to R^2^ = 0.44 (Supplementary Fig. S8). The polarizability, which is the ability to form instantaneous dipoles, of the 19 ILs was positively correlated with the enzyme activity as depicted in Fig. 6c with R^2^ = 0.49 at 24 h of reaction time. Unlike the polarity, the polarizability could account better for the low enzyme activity on [HEMA][MeSO_4_] pretreated cellulose. Removing #2 and #14 ([Apy][Cl] and [TBA][Cl]) improved the fit to R^2^ = 0.79 at 24 h (Supplementary Fig. S8). Of the two series, the anion-series showed the best fit between enzyme activity and Sig3 at 24 h of saccharification with R^2^ = 0.85 compared to R^2^ = 0.26 of the cation-series (Fig. 6d and Supplementary Fig. S7). For the H-bond descriptors, the best fit (R^2^ = 0.93) was observed between H-bond basicity (Hb_acc3) of the anion-series and the activity after 24 h of reaction (Fig. 6e). The fit between activity and Hb_don3 had a R^2^ = 0.54 after 24 h of hydrolysis (Supplementary Fig. S7). This emphasizes that the anion is the determining part when it comes to cellulose solubility, with anions having higher H-bond basicity being better at dissolving cellulose, leading to higher saccharification yields.

## Conclusions

COSMO-RS was used to screen 483 ILs for their ability to solubilize cellulose. COSMO-RS descriptors were then correlated with enzyme activities in the presence of aqueous solutions of ILs of two series at different concentrations. Overall, the correlations were better at the low concentration of IL compared to the intermediate and the high concentration of IL. A clear correlation (R^2^ = 0.88 at 250 mM) was found between the size of the cation and the enzyme activity, with smaller cations having less impact on the activity. Moreover, the predicted logarithmic water activity coefficient of the cation-series correlated negatively with the enzyme activity (R^2^ = 0.86 at 250 mM). In the second part, enzymatic hydrolysis was carried out on IL-pretreated cellulose. Larger negative logarithmic activity coefficient correlated with higher observed enzyme activities with R^2^ = 0.48 for all ILs and R^2^ = 0.75 for the anion-series at 24 h saccharification. Investigation of the interaction energies and the COSMO-RS descriptors revealed that a high H-bond basicity and the H-bond interaction energies between cellulose and the IL were key factors that governed cellulose dissolution and thus enhanced saccharification. Of the experimentally tested ILs, ILs such as [MMIM][DMP], [EMIM][DEP] and [BMIM][Cl] are good candidates to obtain high enzymatic saccharification efficiencies following pretreatment. ILs that were not experimentally tested but were predicted to be good at dissolving cellulose, and thus could be promising, are [HMGua] and [EMpyrr] cations paired with [OAc], [Cl], [DMP] or [DEP] anions. Overall, this work linked molecular descriptors of anion and cations obtained from COSMO-RS with cellulase activity. Additional studies on individual cellulase enzymes may further enhance the correlations between enzyme activity and COSMO-RS based descriptors. This work may lead to the development of a model that integrates the cellulose dissolution predicted by COSMO-RS with ILs σ-profiles or COSMO-RS descriptors impact on cellulase activity for a fast and efficient identification of ILs that promotes cellulose dissolution and are cellulase-friendly at the same time.

## Experimental Section

### Theoretical basis of COSMO-RS

The theory of COSMO-RS has been described by Klamt *et al*.^33–35^. Briefly, COSMO-RS is a quantum based statistical thermodynamic model for the prediction of thermodynamic properties of fluids and liquid mixtures. The quantum chemical basis is a dielectric continuum model called COSMO, where the polarization charge density σ is calculated^36^. The 3D polarization density distribution on the surface of each molecule is converted into a histogram of the screening charge density called a σ-profile (p^x^(σ)), which gives the relative amount of the surface with the polarity σ for a molecule X. The molecular σ-profiles can easily be used to derive the σ-profiles of pure or mixed solvents S using the mole fraction weighted sum of the σ-profiles of its compounds with a surface normalization (Eq. (1))^36^.

1 $${p}_{s}({\rm{\sigma }})=\frac{{\sum }_{i}\,{x}_{i}{p}^{{x}_{i}}({\rm{\sigma }})}{{\sum }_{i}\,{x}_{i}{A}^{{X}_{i}}}$$

Using the screening-charge density, COSMO-RS considers the most relevant molecular interaction modes, electrostatics (E_misfit_) and hydrogen bonding (E_HB_) which are described as functions of the screening charges of two interacting surface segments σ and σ′ or σ_acceptor_ and σ_donor_ (if the segments belongs to the hydrogen bond donor or acceptor atom). As the interactions of the solvent are described by p_s_(σ), the chemical potential of the surface segments is given by Eq. (2) ^36^.

2 $${\mu }_{s}({\rm{\sigma }})=-\frac{RT}{{a}_{eff}}\,\ast \,\mathrm{ln}[\int {p}_{s}(\sigma ^{\prime} )\,\exp (\frac{1}{RT}({a}_{eff}{\mu }_{s}(\sigma ^{\prime} )-{E}_{misfit}(\sigma ,\sigma ^{\prime} )-{E}_{HB}(\sigma ,\sigma ^{\prime} )))d\sigma ^{\prime} ]\,$$

Where µ_s_(σ) is the σ-potential and is a measure of the affinity of the a solvent S for surface of polarity σ. The van der Waal interaction energy (E_vdW_) is not a function of individual surface contacts, but is added to the reference energy in solution *a posteriori*. The chemical potential of compound X_i_ in any pure or mixed solvent S can be calculated by integration of µ_s_(σ) over the surface of the compound (Eq. (3))^36^.

3 $${\mu }_{S}^{X}={\mu }_{{\rm{C}},{\rm{S}}}^{C}+\int {p}^{X}({\rm{\sigma }}){\mu }_{{\rm{s}}}({\rm{\sigma }})d{\rm{\sigma }}$$

Where ${\mu }_{X}^{S}$ denotes the potential of compound X in the reference state of the pure compound and ${\mu }_{{\rm{C}},{\rm{S}}}^{X}$ is an area and volume depending combinatorial term that takes into account the size and shape differences of the molecules in the system. Equation (3) enables the calculation of the chemical potential of all compounds of an arbitrary mixture at a given temperature and a wide variety of thermodynamic properties can be derived e.g. the activity coefficient (Eq. (4))^36^.

4 $${\gamma }_{S}^{X}=\exp (\frac{{\mu }_{S}^{X}-{\mu }_{X}^{S}}{RT})$$

The activity coefficient can be used as a measure for solubility (*x*_*i*_) as the solubility at temperature *T* is expressed as a function of pure component properties of the solute^18^:

5 $$\mathrm{ln}({x}_{i})=\frac{1}{RT}({\mu }_{i}^{i}-{\mu }_{i}^{j})-\,\mathrm{ln}({\gamma }_{i})$$

Where $({\mu }_{i}^{i}-{\mu }_{i}^{j})\,$denotes the free energy difference between the solid state related to its liquid state^18^.

### Computational details and calculations

Some of the studied ILs were present in our in-house database constructed by COSMOlogic. For molecules not in the database, the COSMO-files were generated using TmoleX 16 version 4.2 (COSMOlogic, Leverkusen, Germany). The molecules were first sketched as two-dimensional structures in TMoleX, converted to SMILES annotations and then converted to three-dimensional structures in TMoleX. The geometries of the molecules were optimized at the AM1/COSMO level using the built-in MOPAC program. The polarization charge densities (σ) of the molecular surfaces were then calculated in TmoleX utilizing the BP (B88-VWN-P86) density functional theory (DFT) level with a triple-ζ valence polarized basis set (TZVP). In total, 21 cations and 23 anions were used (483 ILs) (Tables S1 and S2).

### Prediction of logarithmic activity coefficients

The mid-monomer part of cellotriose (Fig. S1) was used as a model to represent cellulose polymer in the COSMO calculations. The COSMO-RS calculations were carried out using the COSMOthermX software (version 16.0.0 applied with parameterization BP_TZVP_C30_1601, COSMOlogic, Leverkusen, Germany). The logarithmic activity coefficient, ln(γ), was calculated at infinite dilution at 90 °C (the same temperature as used for the pretreatment) with the IL ions treated as two different compounds in an equimolar mixture i.e. n_cation_ = n_anion_ = n_IL_.

### Calculation of logarithmic activity coefficient of water and water activities

The logarithmic activity coefficients of water, ln(γ_w_), at 250, 500 and 750 mM IL were calculated for the ILs used for the experimental part using COSMO-RS. This was done by estimating the volume of the ionic liquids based on the mass and density with experimental determined values from the NIST IL database or estimated densities. The molar fraction of water was calculated based on the volume occupied by the IL. The activity coefficient of water was then predicted based on the molar fractions.

## Materials and Methods

### Materials

Cellulase from *Trichoderma reesei* ATCC26921 (>5 units/mg) and Avicel PH-101 (~50 µm particle size) cellulose were purchased from Sigma Aldrich. [AMIM][Cl], [BMIM][DBP], [EMIM][DEP], [Apy][Cl], [HOEMIM][Br], [HOEMIM][Cl] were synthesized following procedures previously reported (See Supporting Information; SI). The synthesized ILs were characterized by ^1^H-NMR on a Bruker 400 spectrometer. The other ILs were purchased from commercial suppliers with purities ranging from >95% to >98% (Table S3). All additional chemicals were from Sigma Aldrich and of analytical purity.

### Saccharification in aqueous solutions of ionic liquids

Stock solutions of ILs were prepared in 50 mM sodium acetate pH 5 and the pH was adjusted to pH 5. Saccharification of the Avicel cellulose without prior pretreatment was carried out in 50 mM sodium acetate buffer pH 5 with IL concentrations of 250, 500 and 750 mM. The Avicel cellulose loading was 2.5% (w/v) and the enzyme concentration was 1 mg mL^−1^ cellulase with a total volume of 1 mL. Reactions were carried out in 2 mL microtubes and incubated in a shaker incubator at 50 °C and 450 RPM for 24 hrs. The reducing sugars were quantified using the DNS-assay as outlined by Ghose^37^ using glucose for the standard curve. The saccharifications were carried out in triplicates. Appropriate blanks were used as controls.

### Pretreatment of cellulose in ILs and subsequent saccharification

As water can inhibit the cellulose dissolution process, all ILs were dried prior to use in a desiccator at 20 mbar for at least 72 hrs. Avicel cellulose was added to the ionic liquids resulting in a 10% (w/w) solution. The pretreatments were carried out in glass tubes with screw caps with magnetic stir bars at 90 °C for 24 hrs at 500 RPM. 100 mg of each ionic liquid/cellulose mixture was then transferred to 2 ml conical bottom microtubes, taking care to mix the cellulose before withdrawing the samples if not dissolved completely in the ILs. 50 mM sodium acetate buffer pH 5 and cellulase enzyme was added to a final volume of 1 mL and 1 mg mL^−1^ enzyme resulting in a 1.0% w/v cellulose and 9.0% w/v ILs mixture. For comparison, mixtures containing 1.0% w/v cellulose and 9.0% w/v IL were incubated without prior pretreatment with 1 mg mL^−1^ cellulase in a total volume of 1 mL. The tubes were incubated in a shaker incubator at 50 °C and 450 RPM. Samples were taken out after 1 h and 24 h incubation. The reducing sugars were quantified using the DNS-assay as outlined by Ghose^37^ using glucose for the standard curve. The saccharifications were carried out in triplicates and appropriate blanks were included.

## Supplementary information

Supplementary Information

## Data Availability

All data generated or analysed during this study are included in this published article (and its Supplementary Information files).
