# Supplementary material for: Stability of cellulase in ionic liquids: correlations between enzyme activity and COSMO-RS descriptors
Source: Sci Rep. 2019 Nov 25;9:17479. doi: 10.1038/s41598-019-53523-5 (PMC6877754; doi:10.1038/s41598-019-53523-5)

**Supporting information**

**Stability of cellulase in ionic liquids: correlations between enzyme activity and COSMO-RS descriptors**

Jacob Nedergaard Pedersen,^1^ Bianca Peréz,^1,2^ Zheng Guo^1*^

^1^Department of Engineering, Faculty of Science and Technology, Aarhus University, 8000, Aarhus, Denmark.

^2^Center for Food Technology, Danish Technological Institute, 8000, Aarhus, Denmark.

*Corresponding author: Zheng Guo; Gustav Wieds vej 10, 8000, Aarhus, Denmark, email: [guo@eng.au.dk](mailto:guo@eng.au.dk).

**Figure S1.** COSMO-RS charge surface of the mid-monomer part of cellotriose.


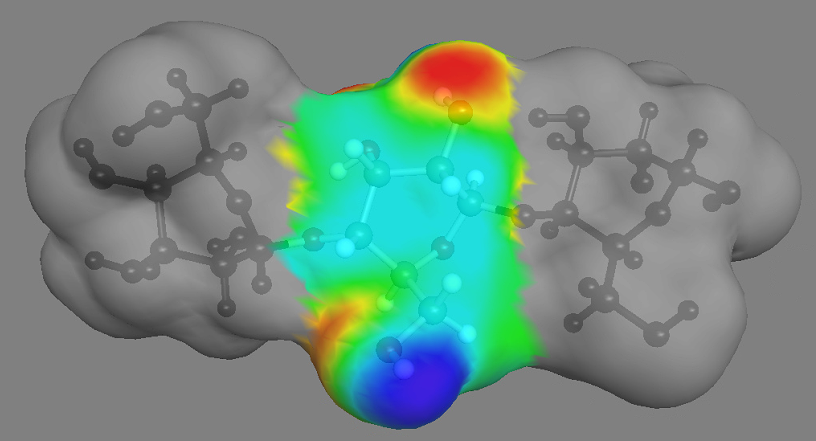

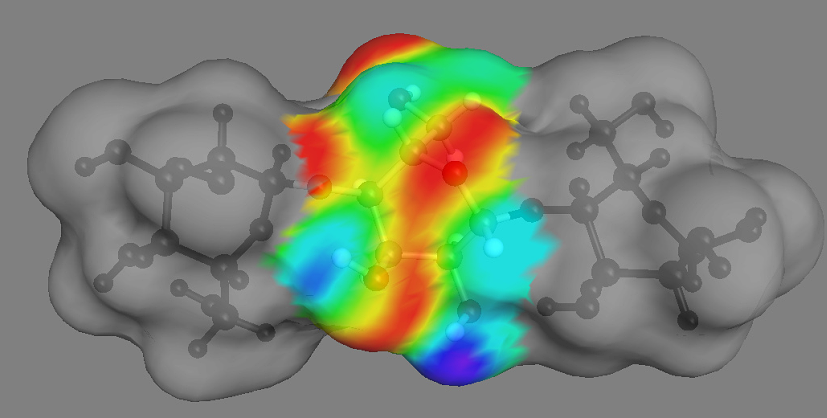


**Table S1.** List of anions used for the COSMO-RS predictions.

| # | Name | Abbreviation | Area [Å^2^] | Sigma2 | Hb_acc3 | Volume [Å^3^] |
| --- | --- | --- | --- | --- | --- | --- |
| 1 | Formate | [HCOO] | 69.81 | 187.38 | 35.11 | 50.83 |
| 2 | Acetate | [OAc] | 90.2 | 204.42 | 38.97 | 72.30 |
| 3 | Butyrate | [Bu] | 108.9 | 201.81 | 38.23 | 93.49 |
| 4 | Decanoate | [Dec] | 248.02 | 207.86 | 38.28 | 247.03 |
| 5 | Chloride | [Cl] | 52.81 | 189.36 | 36.63 | 36.09 |
| 6 | Bromide | [Br] | 58.63 | 170.56 | 29.64 | 42.21 |
| 7 | Iodide | [I] | 67.64 | 147.85 | 18.84 | 52.31 |
| 8 | Dimethylphospate | [DMP] | 142.97 | 214.85 | 35.84 | 131.65 |
| 9 | Diethylphosphate | [DEP] | 180.61 | 207.73 | 34.83 | 175.35 |
| 10 | Dibutylphosphate | [DBP] | 247.91 | 202.48 | 34.05 | 263.07 |
| 11 | Methylsulfate | [MeSO_4_] | 117.89 | 161.46 | 18.5 | 103.86 |
| 12 | Ethylsulfate | [EtSO_4_] | 138.62 | 163.57 | 19.2 | 125.18 |
| 13 | Methanesulfate | [MeSO_3_] | 107.78 | 194.23 | 30.24 | 92.67 |
| 14 | 2(2-methoxyethoxy)ethylsulfate | [MEEtSO_4_] | 168.64 | 183.06 | 19.89 | 214.38 |
| 15 | Methoxyethylsulfate | [MeOEtSO_4_] | 219.67 | 202.88 | 20.76 | 159.39 |
| 16 | Tosylate | [TSO] | 186.61 | 187.66 | 25.77 | 187.22 |
| 17 | Tetrafluoroborate | [BF_4_] | 90.53 | 112.02 | 2.5 | 72.83 |
| 18 | Hexaflurophosphate | [PF_6_] | 114.49 | 88.1 | 0 | 103.68 |
| 19 | Perchlorate | [ClO_4_] | 94.95 | 112.88 | 3.76 | 77.14 |
| 20 | Dicyanamide | [DCA] | 101.7 | 132.97 | 15.53 | 82.43 |
| 21 | Bis(trifluoromethyl)imide | [BTFM] | 132.31 | 86.17 | 4.06 | 122.14 |
| 22 | Tris(nonfluorobutyl)trifluorophosphate | [TNFBTP] | 418.99 | 54.25 | 0.07 | 546.54 |
| 23 | Bis(trifluoromethylsulfonyl)imide | [Tf_2_N] | 203.75 | 97.9 | 1.95 | 219.40 |

**Table S2.** List of cations used for the COSMO-RS predictions.

| # | Name | Abbreviation | Area [Å^2^] | sig 2 | HB_don3 | Volume  [Å^3^] |
| --- | --- | --- | --- | --- | --- | --- |
| 1 | 1,3-dimethylimidazolium | [MMIM] | 143.60 | 88.03 | 2.07 | 133.83 |
| 2 | 1-ethyl-3-methylimidazolium | [EMIM] | 161.77 | 85.38 | 2.07 | 155.78 |
| 3 | 1-butyl-3-methylimidazolium | [BMIM] | 201.97 | 84.71 | 2.06 | 199.85 |
| 4 | 1-allyl-3-methylimidazolium | [AMIM] | 173.60 | 97.63 | 2.31 | 172.36 |
| 5 | 1-hexyl-3-methylimidazolium | [HMIM] | 242.04 | 85.34 | 1.93 | 243.82 |
| 6 | 1-2-hydroxylethyl-3-methylimidazolium | [HOEtMIM] | 172.96 | 131.01 | 5.00 | 167.17 |
| 7 | 1-2-methoxyethyl-3-methylimidazolium | [EtOMIM] | 190.34 | 114.54 | 2.82 | 189.12 |
| 8 | 1-ethylpyridinium | [Epy] | 156.10 | 85.31 | 1.30 | 151.28 |
| 9 | 1-butylpyridinium | [Bpy] | 192.05 | 83.93 | 1.33 | 194.10 |
| 10 | 1-allylpyridinium | [Apy] | 171.06 | 91.89 | 1.27 | 168.15 |
| 11 | 1-butyl-3-methylpyridinium | [BMpy] | 214.56 | 77.76 | 1.07 | 216.58 |
| 12 | 1-2-hydroxylethyl-pyridinium | [HOEtpy] | 167.01 | 130.88 | 4.52 | 162.67 |
| 13 | 1-ethyl-1-methylpyrrolidium | [EMpyrr] | 164.08 | 73.83 | 0.07 | 171.18 |
| 14 | 1-butyl-1-methylpyrolidium | [BMpyrr] | 203.90 | 73.14 | 0.06 | 215.14 |
| 15 | 1-allyl-1-methylpyrrolidium | [AMpyrr] | 178.90 | 79.94 | 0.05 | 188.49 |
| 16 | 1-hexyl-1-methylpyrrolidium | [HMpyrr] | 243.82 | 74.36 | 0.06 | 259.21 |
| 17 | Hexamethylguandium | [HMGua] | 197.01 | 58.66 | 0.00 | 215.32 |
| 18 | Tetrabutylphosphonium | [TBP] | 349.44 | 69.10 | 0.02 | 396.29 |
| 19 | Trihexyltetradecylphosphonium | [TTP] | 668.07 | 81.25 | 0.02 | 748.11 |
| 20 | Tetrabutylammonium | [TBA] | 335.68 | 67.98 | 0.09 | 376.14 |
| 21 | Tris-(2-hydroxyethyl)-methylammonium | [HEMA] | 195.47 | 182.35 | 8.32 | 215.08 |

**Table S3.** Ionic liquids used for experimental verification in this study.

|  | Abbreviation | Cation | Anion | Supplier | Purity |
| --- | --- | --- | --- | --- | --- |
| 1 | [AMIM][Cl] | 1-allyl-3-methylimidazolium | Chloride | Synthesized |  |
| 2 | [Apy][Cl] | 1-allylpyridinium | Chloride | Synthesized |  |
| 3 | [BMIM][BF_4_] | 1-butyl-3-methylimidazolium | Tetrafluoroborate | Merck Solvent Innovation GmbH | >98% |
| 4 | [BMIM][Cl] | 1-butyl-3-methylimidazolium | Chloride | Sigma Aldrich | >98% |
| 5 | [BMIM][DBP] | 1-butyl-3-methylimidazolium | Dibutylphosphate | Synthesized |  |
| 6 | [BMIM][MeSO_4_] | 1-butyl-3-methylimidazolium | Methylsulfate | Sigma Aldrich | >98% |
| 7 | [EMIM][Br] | 1-ethyl-3-methylimidazolium | Bromide | Sigma Aldrich | >97% |
| 8 | [EMIM][Cl] | 1-ethyl-3-methylimidazolium | Chloride | Sigma Aldrich | >95% |
| 9 | [EMIM][DEP] | 1-ethyl-3-methylimidazolium | Diethylphosphhate | Synthesized |  |
| 10 | [EMIM][I] | 1-ethyl-3-methylimidazolium | Iodide | abcr | >95% |
| 11 | [EMIM][OAc] | 1-ethyl-3-methylimidazolium | Acetate | Abcr | >95% |
| 12 | [EMIM][TSO] | 1-ethyl-3-methylimidazolium | Tosylate | Merck Solvent Innovation GmbH | >98% |
| 13 | [EtOMIM][Cl] | 1-2-methoxyethyl-3-methylimidazolium | Chloride | Synthesized |  |
| 14 | [HEMA][MeSO_4_] | Tris(2-hydroxyethyl)methylammonium | Methylsulfate | Sigma Aldrich | >95% |
| 15 | [HMIM][Cl] | 1-hexyl-3-methylimidazolium | Chloride | Merck Solvent Innovation GmbH | >98% |
| 16 | [HOEtMIM][Br] | 1-(2-hydroxylethyl)-3-methylimidazolium | Bromide | Synthesized |  |
| 17 | [HOEtpy][Br] | 1-(2-hydroxyethyl)-pyridinium | Bromide | Synthesized |  |
| 18 | [MMIM][DMP] | 1-methyl-3-methylimidazolium | Dimethylphosphate | Merck Solvent Innovation GmbH | >98% |
| 19 | [TBA][Cl] | Tetrabutylammonium | Chloride | Sigma Aldrich | >97% |

**Figure S2.** Tabulated values of the Predicted logarithmic activity coefficients of the mid-monomer part of cellotriose in 483 ILs at infinite dilution. The calculation temperature was 90 ^o^C.

| **[TNFBTP]** | 1.93 | 1.54 | 1.55 | 1.62 | 2.02 | 1.67 | 2.05 | 1.57 | 1.22 | 1.32 | 1.33 | 1.40 | 1.45 | 1.41 | 0.94 | 1.34 | 1.22 | 2.30 | 0.61 | 0.62 | 0.17 |
| --- | --- | --- | --- | --- | --- | --- | --- | --- | --- | --- | --- | --- | --- | --- | --- | --- | --- | --- | --- | --- | --- |
| **[Tf_2_N]** | 0.14 | 0.09 | 0.15 | 0.13 | 0.49 | 0.20 | 0.53 | 0.22 | 0.19 | 0.16 | 0.20 | 0.17 | 0.21 | 0.19 | 0.07 | 0.27 | 0.23 | 1.11 | 0.05 | 0.11 | 0.00 |
| **[PF_6_]** | 0.08 | 0.25 | 0.33 | 0.18 | 0.48 | 0.21 | 0.53 | 0.23 | 0.68 | 0.41 | 0.53 | 0.22 | 0.19 | 0.29 | 0.34 | 0.59 | 0.55 | 1.21 | 0.52 | 0.67 | 0.63 |
| **[BTFM]** | -0.84 | -0.58 | -0.52 | -0.65 | -0.42 | -0.61 | -0.39 | -0.47 | -0.15 | -0.32 | -0.25 | -0.45 | -0.45 | -0.40 | -0.27 | -0.19 | -0.20 | 0.30 | 0.00 | 0.10 | 0.15 |
| **[ClO_4_]** | -1.01 | -0.71 | -0.49 | -0.58 | -0.32 | -0.49 | -0.27 | -0.43 | -0.08 | -0.26 | -0.12 | -0.34 | -0.35 | -0.27 | -0.15 | 0.07 | 0.09 | 0.47 | 0.19 | 0.43 | 0.72 |
| **[BF_4_]** | -1.68 | -1.34 | -1.09 | -1.16 | -0.88 | -1.04 | -0.83 | -0.96 | -0.68 | -0.82 | -0.66 | -0.86 | -0.85 | -0.77 | -0.63 | -0.43 | -0.42 | 0.00 | -0.24 | 0.03 | 0.45 |
| **[MEEtSO_4_]** | -2.58 | -2.20 | -2.06 | -2.10 | -1.93 | -1.99 | -1.90 | -1.89 | -1.77 | -1.84 | -1.77 | -1.88 | -1.73 | -1.75 | -1.65 | -1.63 | -1.58 | -1.30 | -1.38 | -1.27 | -0.90 |
| **[MeOEtSO_4_]** | -2.82 | -2.39 | -2.21 | -2.24 | -2.05 | -2.11 | -2.02 | -2.00 | -1.88 | -1.95 | -1.86 | -1.79 | -1.82 | -1.83 | -1.72 | -1.68 | -1.64 | -1.25 | -1.41 | -1.27 | -0.83 |
| **[EtSO_4_]** | -3.03 | -2.54 | -2.32 | -2.36 | -2.17 | -2.22 | -2.14 | -2.08 | -1.93 | -2.01 | -1.90 | -1.94 | -1.88 | -1.88 | -1.75 | -1.70 | -1.65 | -1.39 | -1.38 | -1.21 | -0.73 |
| **[MeSO_4_]** | -3.10 | -2.63 | -2.36 | -2.37 | -2.15 | -2.21 | -2.11 | -2.06 | -1.98 | -2.02 | -1.90 | -1.91 | -1.84 | -1.84 | -1.73 | -1.65 | -1.60 | -1.35 | -1.35 | -1.15 | -0.64 |
| **[DCA]** | -3.19 | -2.57 | -2.34 | -2.46 | -2.39 | -2.36 | -2.37 | -1.97 | -1.50 | -1.72 | -1.51 | -1.79 | -1.81 | -1.65 | -1.45 | -1.30 | -1.31 | -1.60 | -0.88 | -0.59 | -0.22 |
| **[I]** | -3.48 | -3.18 | -2.76 | -2.56 | -2.19 | -2.26 | -2.15 | -2.05 | -2.60 | -2.31 | -2.16 | -1.91 | -1.76 | -1.80 | -1.89 | -1.72 | -1.76 | -1.31 | -1.67 | -1.42 | -0.93 |
| **[TSO]** | -3.62 | -3.02 | -2.85 | -2.93 | -2.87 | -2.83 | -2.85 | -2.61 | -2.35 | -2.47 | -2.36 | -2.45 | -2.41 | -2.38 | -2.21 | -2.20 | -2.14 | -2.19 | -1.80 | -1.66 | -1.20 |
| **[MeSO_3_]** | -4.91 | -4.28 | -3.94 | -3.87 | -3.64 | -3.62 | -3.61 | -3.42 | -3.58 | -3.49 | -3.36 | -3.23 | -3.09 | -3.15 | -3.06 | -3.04 | -2.99 | -2.84 | -2.66 | -2.46 | -1.77 |
| **[Br]** | -5.11 | -4.70 | -4.21 | -3.90 | -3.49 | -3.49 | -3.45 | -3.21 | -4.13 | -3.66 | -3.53 | -3.05 | -2.82 | -2.93 | -3.09 | -3.02 | -3.05 | -2.61 | -2.90 | -2.71 | -2.09 |
| **[DBP]** | -5.14 | -4.26 | -4.11 | -4.21 | -4.34 | -4.13 | -4.34 | -3.84 | -3.53 | -3.63 | -3.54 | -3.61 | -3.57 | -3.55 | -3.32 | -3.41 | -3.31 | -3.74 | -2.81 | -2.71 | -2.00 |
| **[DEP]** | -5.52 | -4.57 | -4.37 | -4.46 | -4.55 | -4.35 | -4.55 | -4.05 | -3.74 | -3.85 | -3.73 | -3.80 | -3.75 | -3.72 | -3.49 | -3.55 | -3.45 | -3.90 | -2.93 | -2.79 | -2.04 |
| **[DMP]** | -5.74 | -4.83 | -4.56 | -4.61 | -4.60 | -4.44 | -4.59 | -4.14 | -3.97 | -4.02 | -3.89 | -3.90 | -3.82 | -3.82 | -3.61 | -3.65 | -3.56 | -3.89 | -3.07 | -2.89 | -2.12 |
| **[Cl]** | -6.60 | -6.01 | -5.46 | -5.10 | -4.67 | -4.63 | -4.63 | -4.38 | -5.46 | -4.91 | -4.80 | -4.19 | -3.90 | -4.09 | -4.24 | -4.25 | -4.25 | -3.80 | -4.03 | -3.89 | -3.12 |
| **[HCOO]** | -7.35 | -6.31 | -5.86 | -5.72 | -5.62 | -5.40 | -5.61 | -4.98 | -5.33 | -5.09 | -4.94 | -4.69 | -4.53 | -4.57 | -4.47 | -4.51 | -4.43 | -4.87 | -3.91 | -3.71 | -2.72 |
| **[Dec]** | -7.37 | -5.93 | -5.76 | -5.90 | -6.29 | -5.83 | -6.33 | -5.30 | -4.82 | -4.96 | -4.85 | -4.93 | -4.92 | -4.86 | -4.53 | -4.71 | -4.54 | -5.82 | -3.73 | -3.62 | -2.63 |
| **[Bu]** | -8.08 | -6.58 | -6.26 | -6.32 | -6.55 | -6.13 | -6.57 | -5.57 | -5.32 | -5.36 | -5.20 | -5.20 | -5.13 | -5.09 | -4.81 | -4.92 | -4.79 | -5.94 | -3.99 | -3.80 | -2.71 |
| **[OAc]** | -8.25 | -6.85 | -6.47 | -6.44 | -6.56 | -6.19 | -6.57 | -5.66 | -5.64 | -5.57 | -5.41 | -5.30 | -5.19 | -5.18 | -4.97 | -5.06 | -4.95 | -5.90 | -4.22 | -4.02 | -2.94 |
|  | **[HMGua]** | **[EMpyrr]** | **[AMpyrr]** | **[BMpyrr]** | **[TBA]** | **[HMpyrr]** | **[TBP]** | **[BMpy]** | **[MMIM]** | **[EMIM]** | **[Epy]** | **[BMIM]** | **[HMIM]** | **[Bpy]** | **[EtOMIM]** | **[Apy]** | **[AMIM]** | **[TTP]** | **[HOEtMIM]** | **[HOEtpy]** | **[HEMA]** |

**Figure S3**: Effect of 250 mM, 500 mM and 750 mM concentrations of ILs (pH 5) on the cellulase activity relative to buffer (50 mM sodium acetate, pH 5). The cellulose loading was 2.5% (w/v) and the cellulase loading was 1 mg mL^-1^. The cation series is gathered to left and the anion series is gathered to the right.

**
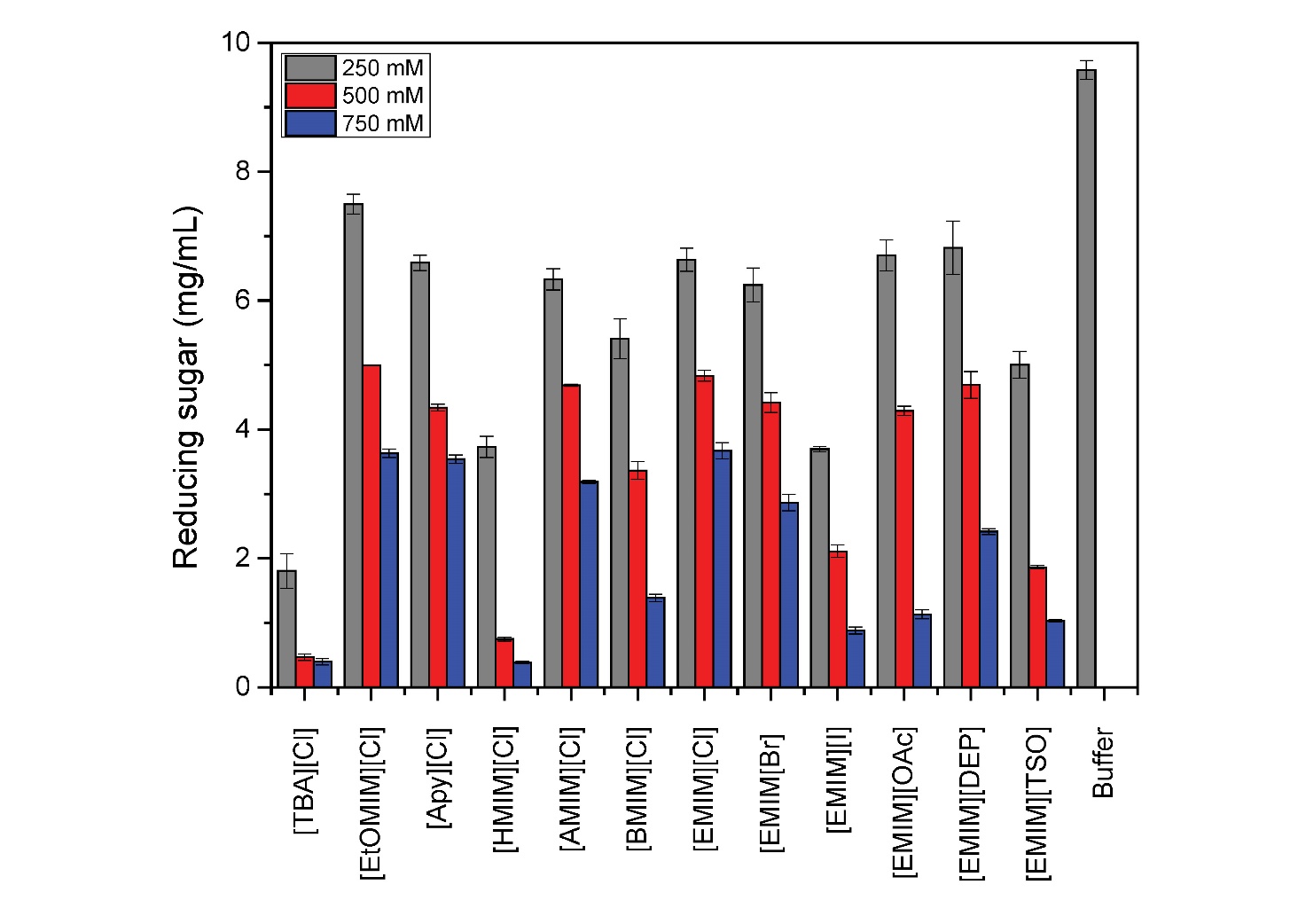
**

**Figure S4.** Amount of reducing sugar released (mg mL^-1^) after 24h of saccharification on untreated cellulose at 250 mM (■), 500 mM (●) and 750 mM (▲) IL versus COSMO-RS molecular descriptors for: a) Sig2 for the anion-seires, b) sig2 for the cation-series c) anion-series area (Å^2^), d) anion-series volume (Å^3^), e) cation-series volume (Å^3^), f) anion-series ln(γ_w_).


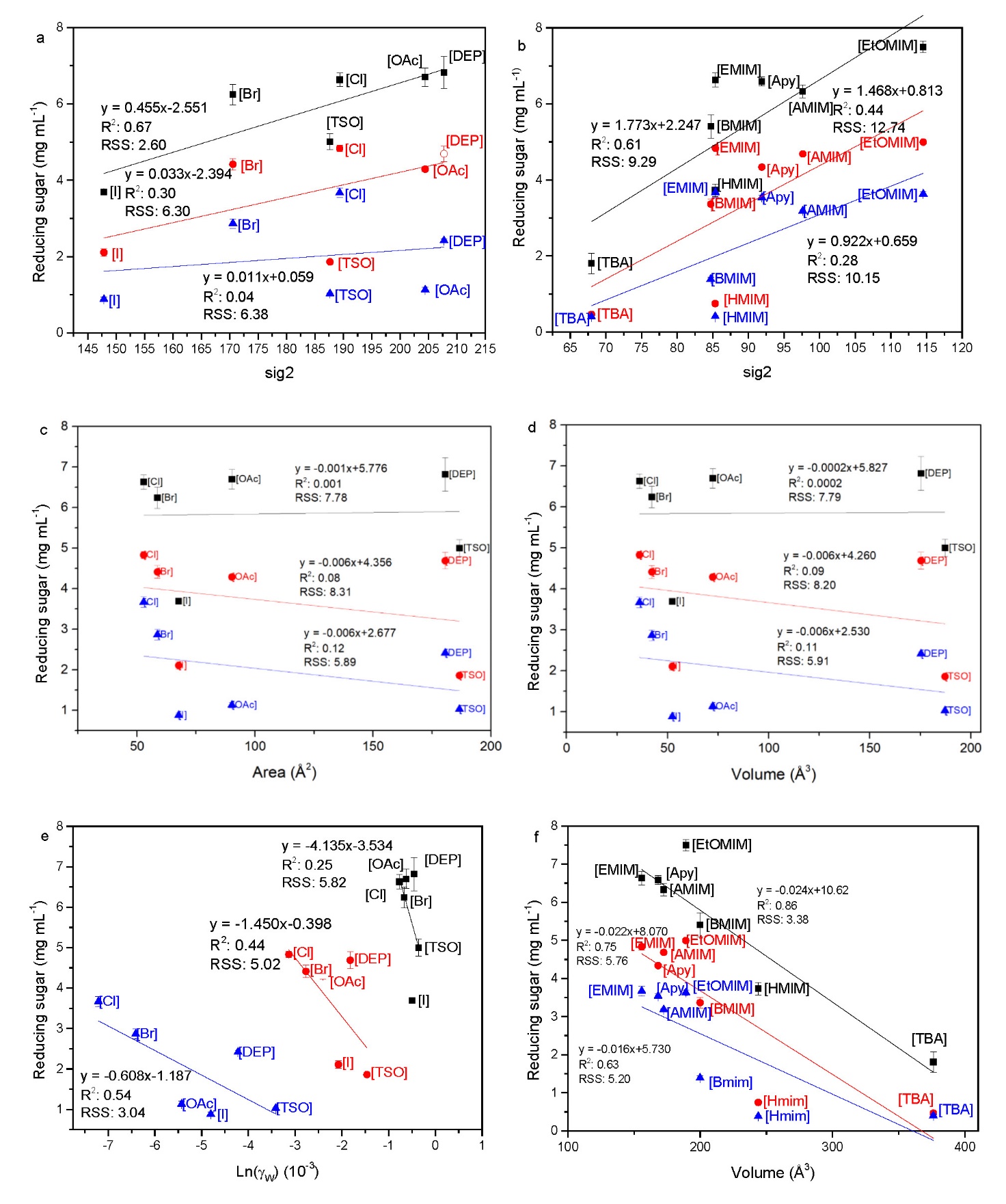


**Figure S5.** Reducing sugar (mg mL^-1^) measured after 1h and 24h of hydrolysis of pretreated cellulose versus different interaction energies of the mid-monomer part of cellotriose at infinite dilution and COSMO-RS descriptors for all ILs: a) van-der-Waal interaction energy (kcal mol^-1^), b) misfit interaction energy (kcal mol^-1^), c) area (Å^2^), d) Volume (Å^3^). Experimental conditions: cellulose loading = 2.5% (w/v), cellulase loading = 1 mg mL^-1^, ILs were dissolved in 50 mM sodium acetate, pH = 5.


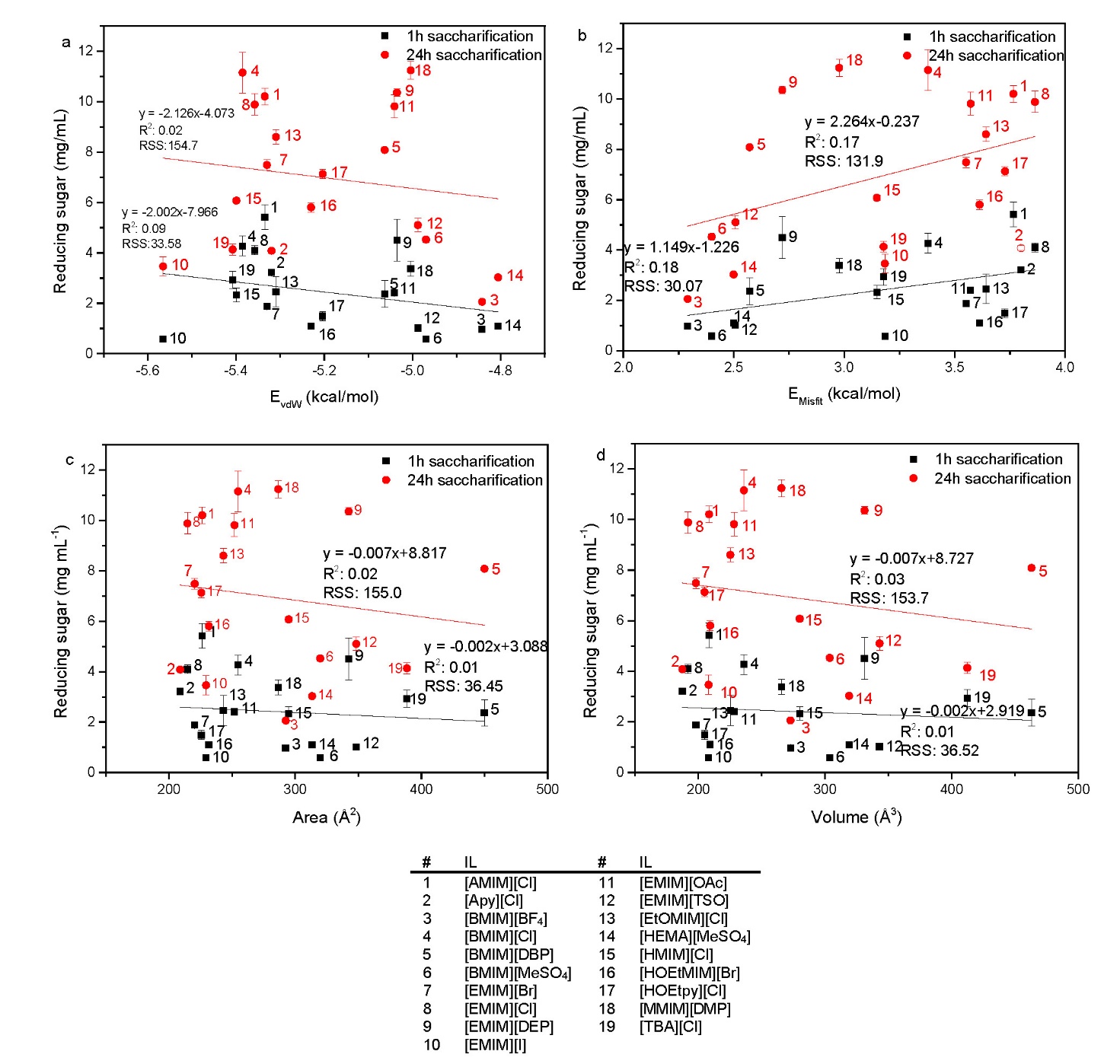


**Figure S6.**  Reducing sugar (mg mL^-1^) measured after 1h and 24h of hydrolysis of pretreated cellulose versus different interaction energies of the mid-monomer part of cellotriose at infinite dilution and COSMO-RS descriptors for the anion-series: a) misfit interaction energy (kcal mol^-1^), b) van der Waal interaction energy (kcal mol^-1^), c) area (Å^2^), d) volume (Å^3^).


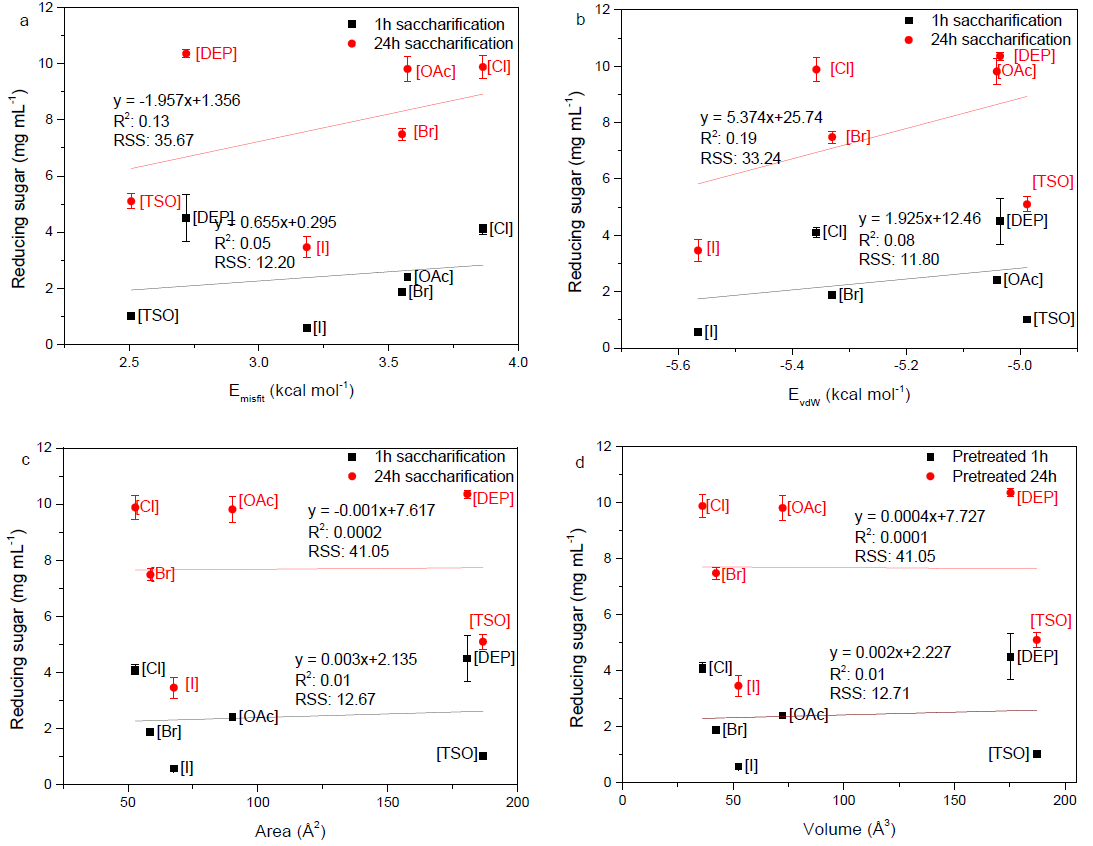


**Figure S7.** Reducing sugar (mg mL^-1^) measured after 1h and 24h of hydrolysis of pretreated cellulose versus different interaction energies of the mid-monomer part of cellotriose at infinite dilution and COSMO-RS descriptors for the cation-series: a) logarithmic activity coefficient, b) hydrogen bonding interaction energy (kcal mol^-1^), c) misfit interaction energy (kcal mol^-1^), d) van der Waal interaction energy (kcal mol^-1^), e) area (Å^2^), f) volume (Å^3^).


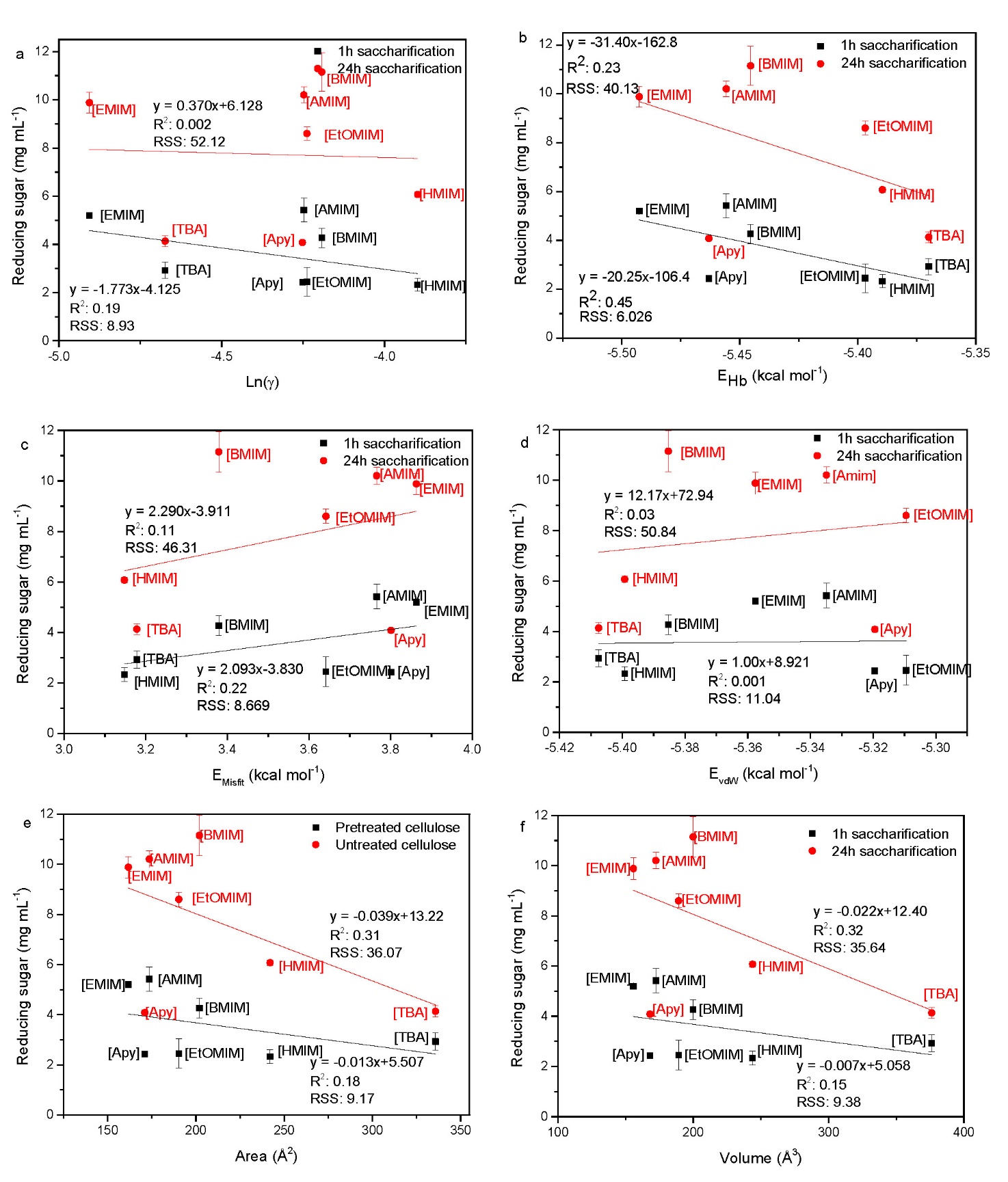


**Figure S8.**  Plots of reducing sugar (mg mL^-1^) measured after 24h of hydrolysis of pretreated cellulose versus different interaction energies of the mid-monomer part of cellotriose at infinite dilution and COSMO-RS descriptors with **r**emoved outliers, a) logarithmic activity coefficient for all ILs with #2 and #19 removed, b) hydrogen bonding interaction energy (kcal mol^-1^) for all ILs with #2 and #19 removed, c) Sig2 for all ILs with #14 removed, d) Sig for all ILs with #2 and #19 removed.

**
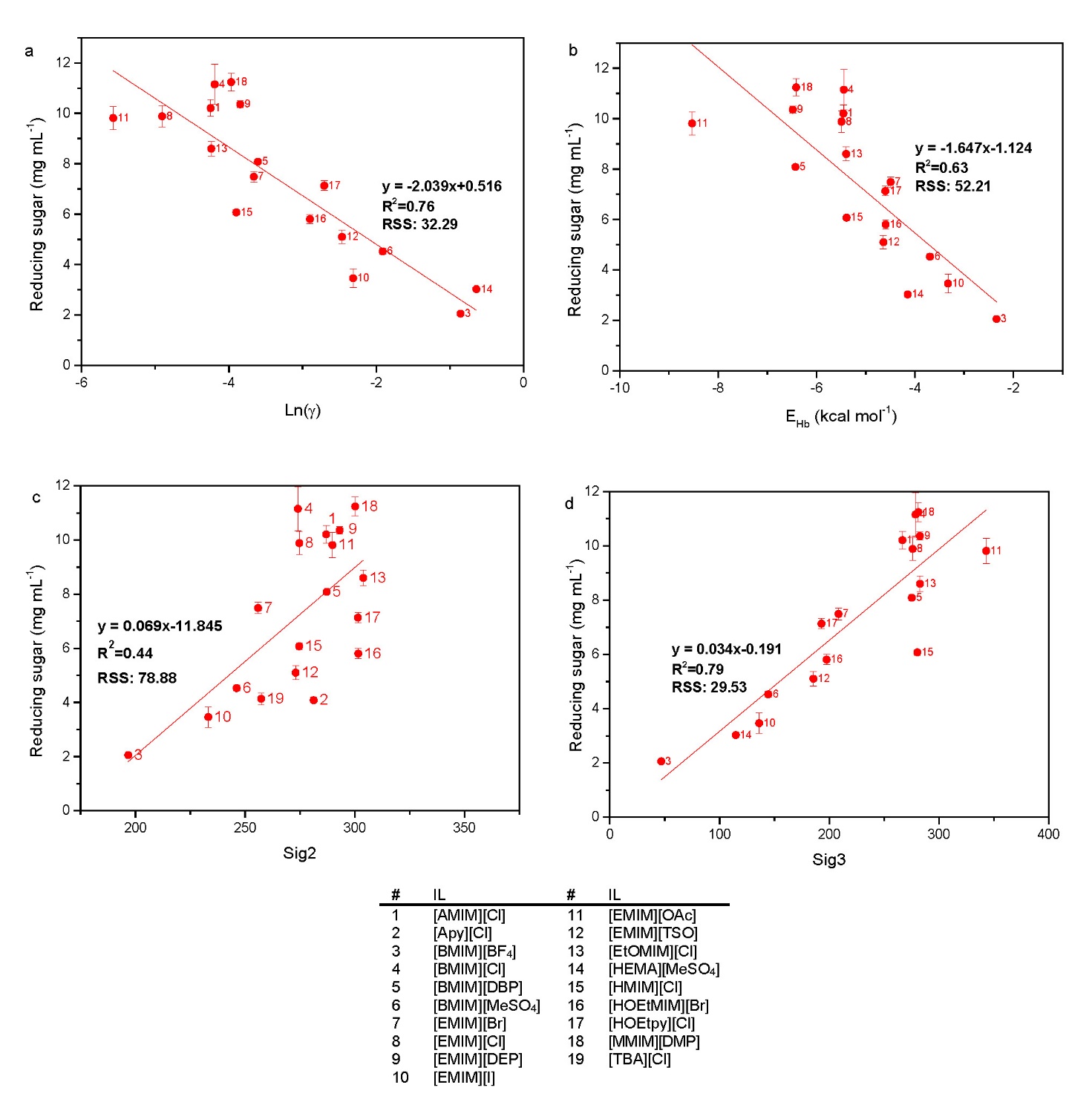
**

**Synthesis of ionic liquids**

**Synthesis of ionic liquids**

[EMIM][DEP] was synthesized according to Liu et al.^1^ with some modifcations. Briefly, 1-methylimidazole (0.15 mol) and triethylphosphate (0.15 mol) was mixed and stirred under reflux at 80 ^o^C for 8 hrs and then stirred at 150 ^o^C for 40 hrs. After cooling to room temperature, the reaction mixture was washed with diethylether (4×30 mL). The product was dried in a rotary evaporator at 75 ^o^C for 2 hrs and then dried under vacuum in a dessicator for 72 hrs to obtain [EMIM][DEP] as a viscous yellow liquid.

Synthesis of [BMIM][DBP]: 1-methylimidazole (0.15 mol) and tributylphosphate (0.15 mol) was mixed and stirred under reflux at 80 ^o^C for 8 hrs and then stirred at 150 ^o^C for 40 hrs. After cooling to room temperature, the reaction mixture was washed with diethylether (4×30 mL). The product was dried in a rotary evaporator at 85 ^o^C for 2 hrs and then dried under vacuum in a dessicator for 72 hrs to obtain [BMIM][DBP] as a viscous yellow/brown liquid.

[AMIM][Cl] was synthesized according to Liu et al.^1^ with some modifications. Briefly, Allyl chloride (0.155 mol) was added slowly to 1-methylimidazole (0.15 mol) in an ice-bath and stirred for 6 hrs under nitrogen protection, then heated under reflux at 60 ^o^C for 24 hrs. The reaction mixture was cooled to room temperature and washed with ethyl acetate (4×30 mL), followed by rotary vacuum evaporation at 80 ^o^C for 2h and then dried under vacuum in a dessicator for 72 hrs to obtain [AMIM][Cl] as a viscous yellow/brown liquid.

[Apy][Cl] was synthesized according to Liu et al.^1^ with some modifications. Allyl chloride (0.20 mol) was dropped slowly into pyridine (0.20 mol) in an ice-bath and stirred for 6h under nitrogen protection, followed by stirring at 60 ^o^C for 24h under reflux. After cooling to room temperature the reaction mixture was washed with ethyl acetate (4×30 mL). The product was dried in a rotary evaporator at 85 ^o^C for 2 hrs and then dried under vacuum in a desiccator for 72 hrs to obtain [APy][Cl] as a brown solid.

[HOEMIM][Br] was synthesized according to Liu et al.^1^ with some modifications. 2-bromoethanol (0.20 mol) was added slowly to 1-methylimidazole (0.19 mol) in an ice-bath and stirred for 6h under nitrogen protection, then heated under reflux at 80 ^o^C for 48h. The reaction mixture was cooled to room temperature and washed with ethyl acetate (4×30 mL), followed by rotary vacuum evaporation at 80 ^o^C for 2h and then dried under vacuum in a dessicator for 72 hrs to obtain [HOEMIM][Br] as a brown solid.

[HOEtPy][Br] was synthesized according to Liu et al.^1^ with some modifications. 2-bromoethanol (0.20 mol) was dropped slowly into pyridine (0.19 mol) in an ice-bath and stirred for 6h under nitrogen protection, followed by stirring at 80 ^o^C for 48h under reflux. After cooling to room temperature the reaction mixture was washed with ethyl acetate (4×30 mL). The product was dried in a rotary evaporator at 85 ^o^C for 2 hrs and then dried under vacuum in a desiccator for 72 hrs to obtain [HOEtPy][Br] as a light brown solid.

[EtOmim][Cl] was synthesized according to Liu et al.^1^ with some modifications. 2-chloroethyl methyl ether (0.20 mol) was added slowly to 1-methylimidazole (0.19 mol) in an ice-bath and stirred for 6h under nitrogen protection, then heated under reflux at 80 ^o^C for 48h. The reaction mixture was cooled to room temperature and washed with ethyl acetate (4×30 mL). Followed by rotary vacuum evaporation at 80 ^o^C for 2h and then dried under vacuum in a desiccator for 72 hrs to obtain [EtOMIM][Cl] as a white solid.

1. Liu, Y.-R., Thomsen, K., Nie, Y., Zhang, S.-J. & Meyer, A. S. Predictive screening of ionic liquids for dissolving cellulose and experimental verification. *Green Chem.* **18,** 6246–6254 (2016).

**NMR-data of the synthesized ILs**

The structures of the synthesized ILs was characterized by ^1^H-NMR-spectroscopy on a Bruker 400 mHz NMR. The data is shown below:

[EMIM][DEP]:


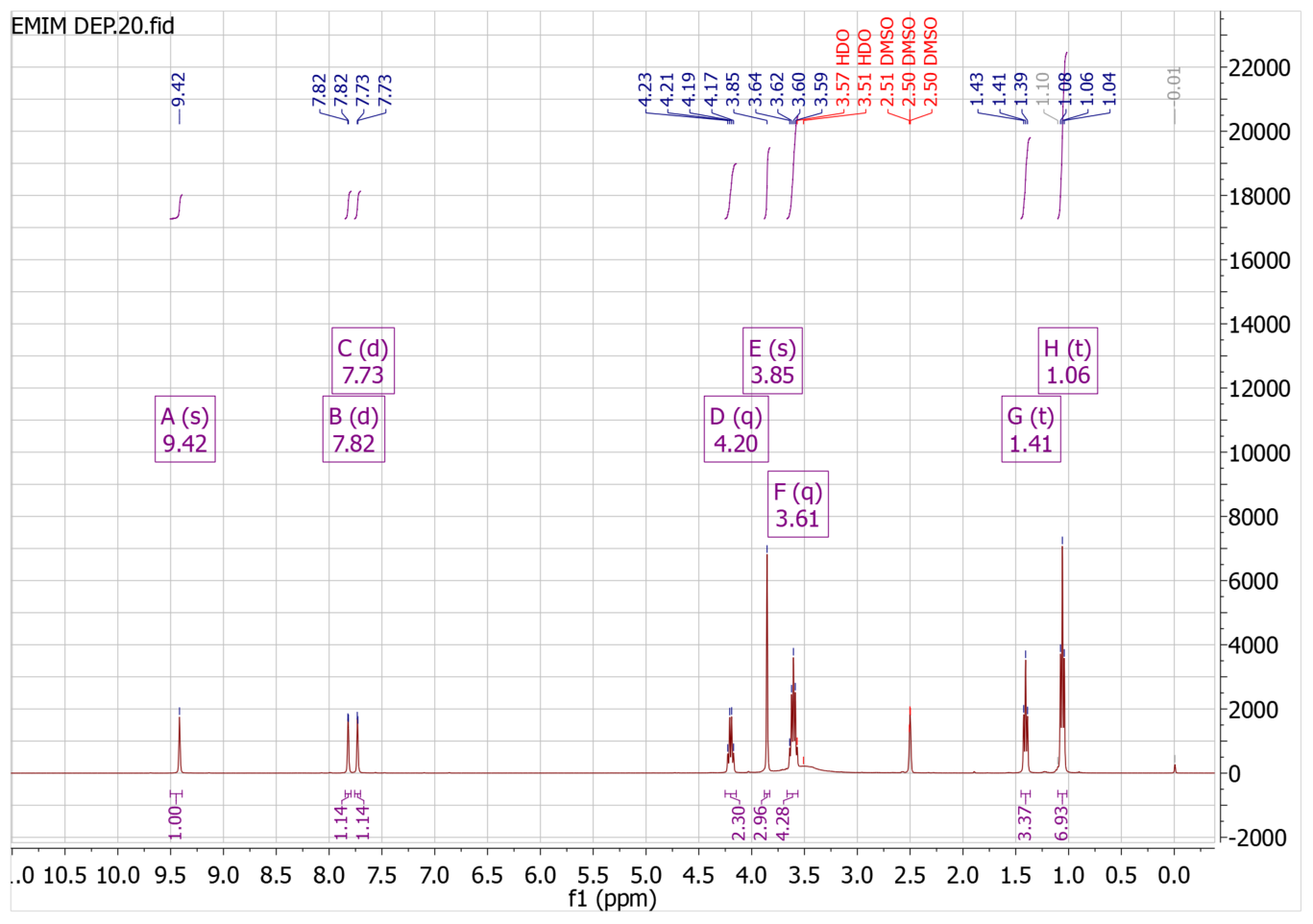


[BMIM][DBP]


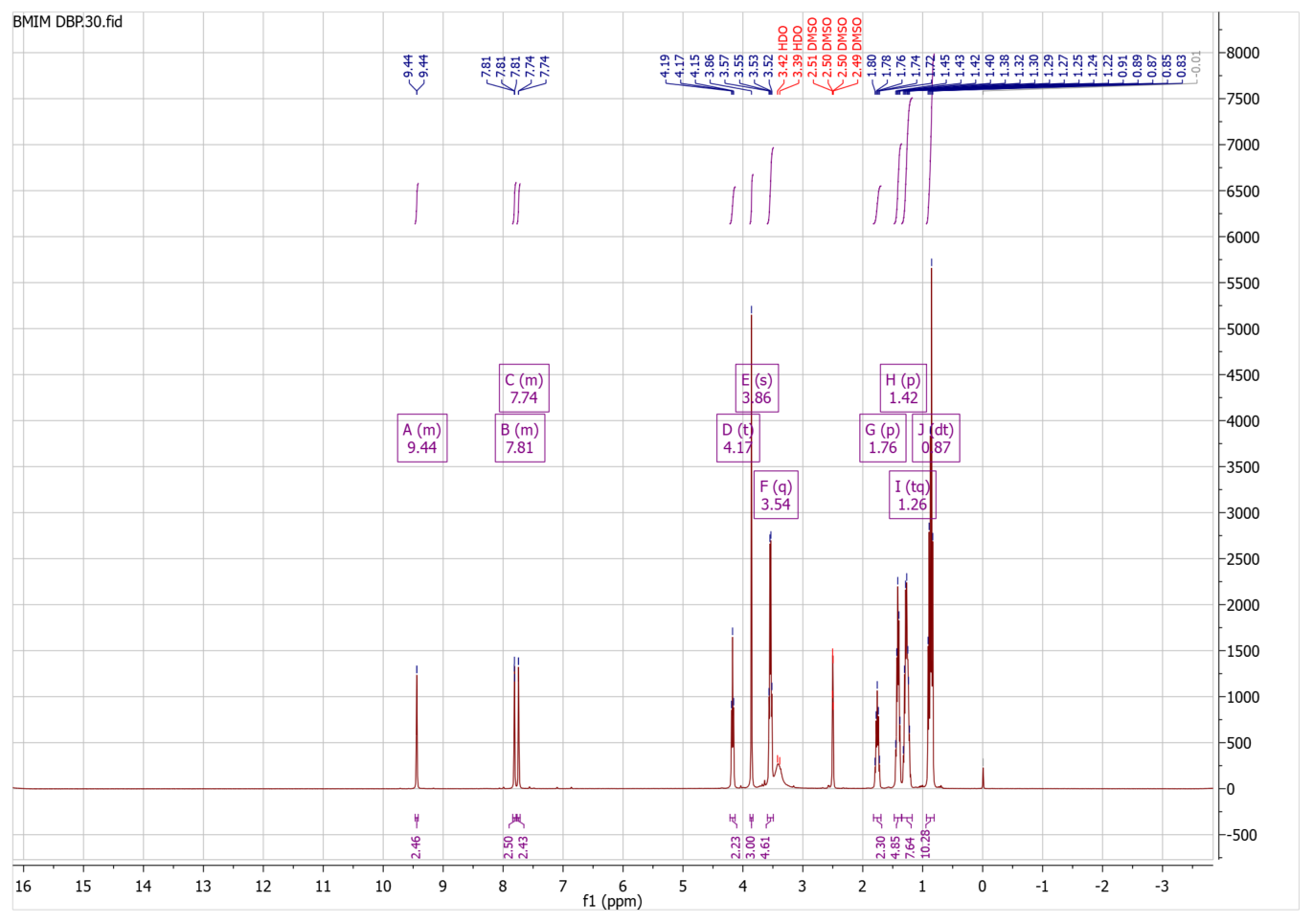


[AMIM][Cl]


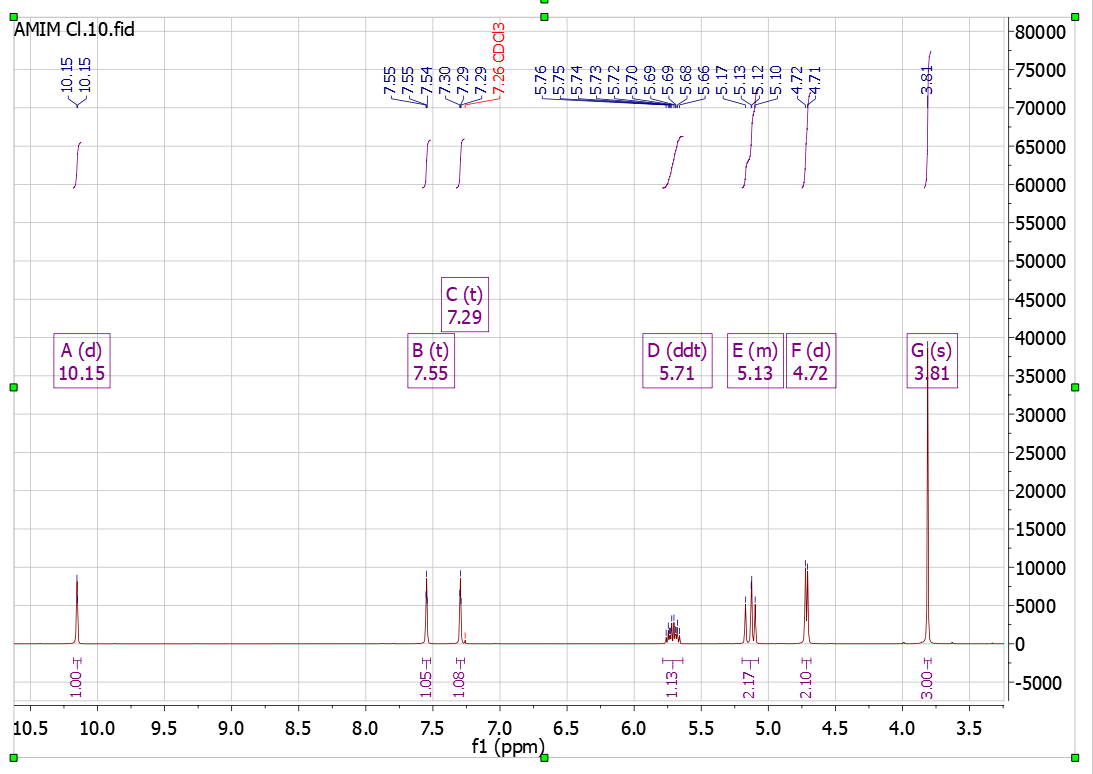


[Apy][Cl]


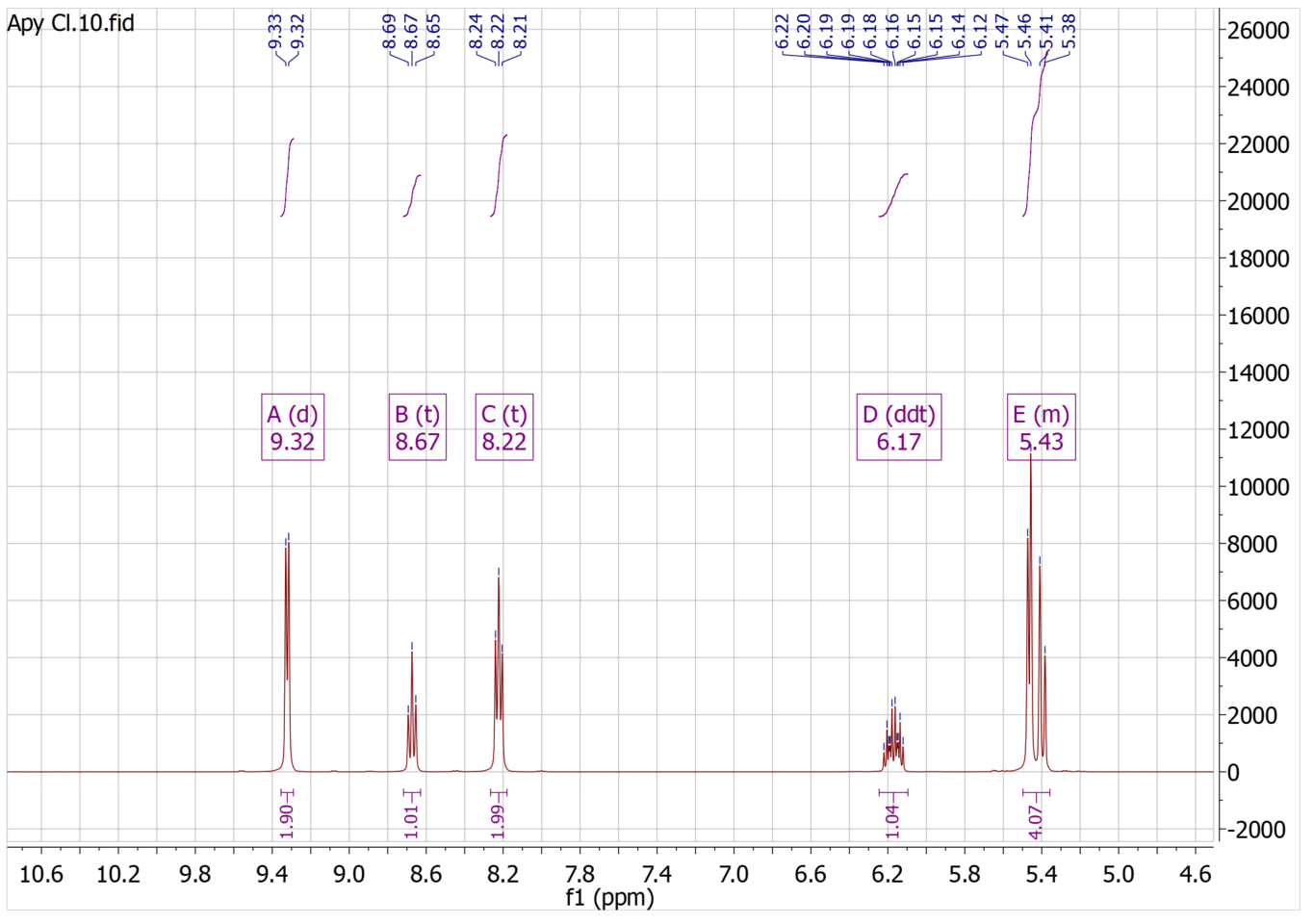


[HOEMIM][Br]


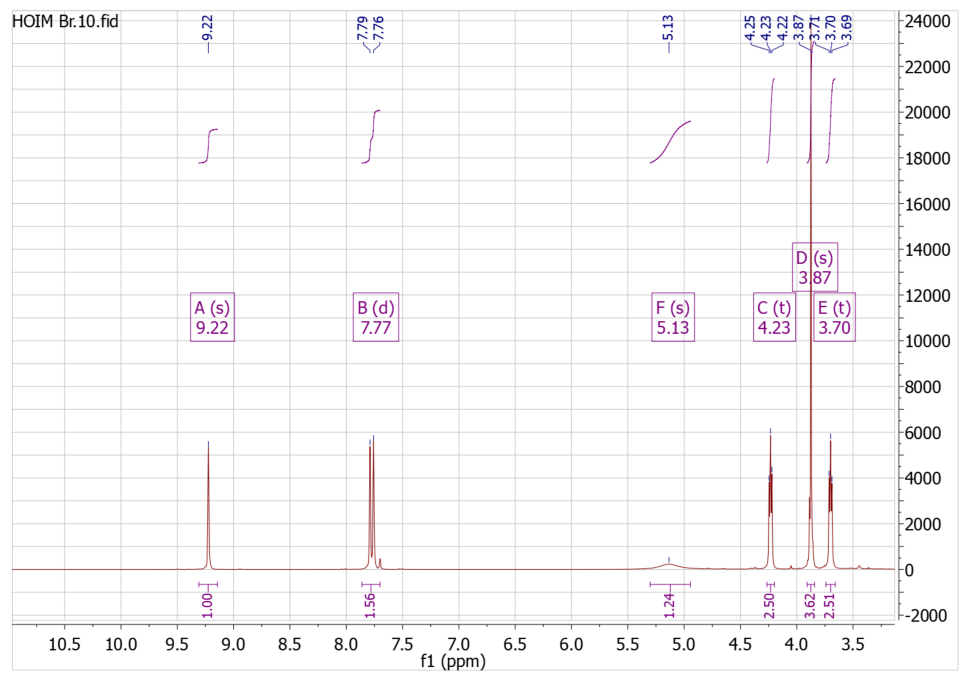


[HOpy][Br]


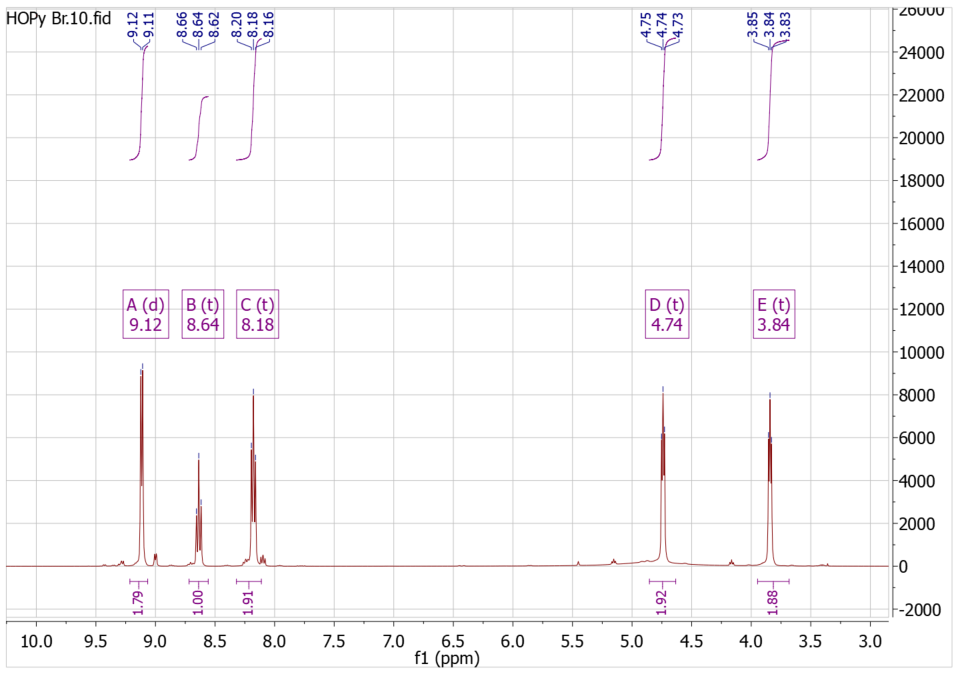


[EtOMIM][Cl]


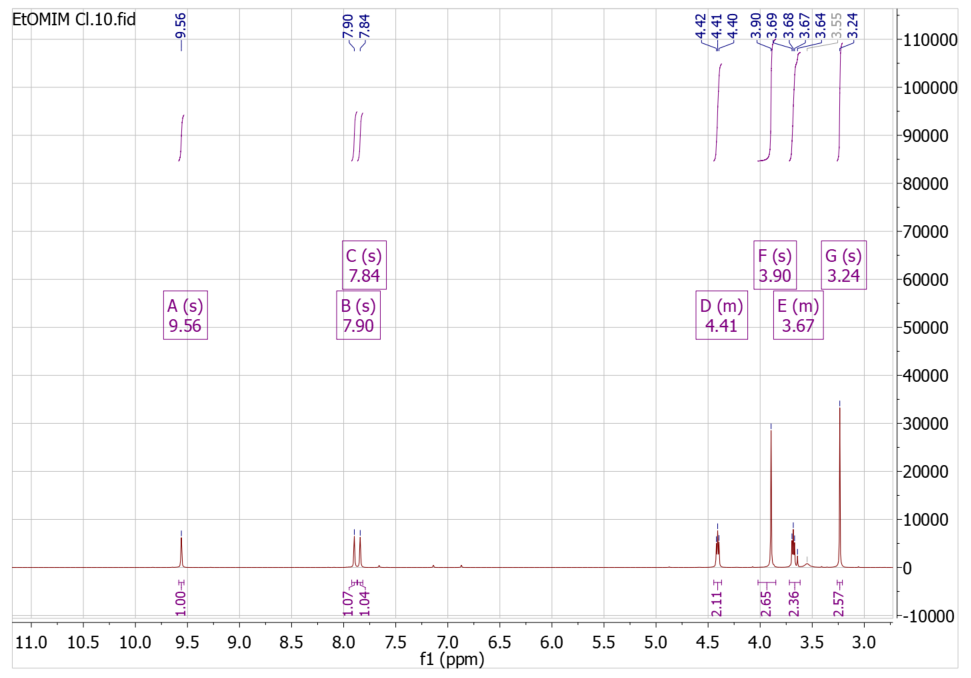

Supplement: Supplementary file 1 — Supplementary Information [file 41598_2019_53523_MOESM1_ESM.docx]
